# Inhibition of protein translational machinery in triple-negative breast cancer as a promising therapeutic strategy

**DOI:** 10.1016/j.xcrm.2024.101552

**Published:** 2024-05-09

**Authors:** Arpit Dheeraj, Fernando Jose Garcia Marques, Dhanir Tailor, Abel Bermudez, Angel Resendez, Mallesh Pandrala, Benedikt Grau, Praveen Kumar, Carrsyn B. Haley, Alexander Honkala, Praveen Kujur, Stefanie S. Jeffrey, Sharon Pitteri, Sanjay V. Malhotra

**Affiliations:** 1 Department of Cell, Developmental and Cancer Biology, Knight Cancer Institute, Oregon Health & Science University, Portland, OR, USA; 2 Center for Experimental Therapeutics, Knight Cancer Institute, Oregon Health & Science University, Portland, OR, USA; 3 Department of Radiation Oncology, Stanford University School of Medicine, Palo Alto, CA, USA; 4 Department of Radiology, Canary Center at Stanford for Cancer Early Detection, Stanford University School of Medicine, Palo Alto, CA, USA; 5 Department of Surgery, Stanford University School of Medicine, Palo Alto, CA, USA

**Keywords:** YB-1, protein translation, ribosomes, RPL, RPS, small molecule, eIF, CETSA, proteosome profiling, triple-negative breast cancer

## Abstract

Y-box binding protein-1 (YB-1) is a proto-oncogenic protein associated with protein translation regulation. It plays a crucial role in the development and progression of triple-negative breast cancer (TNBC). In this study, we describe a promising approach to inhibit YB-1 using SU056, a small-molecule inhibitor. SU056 physically interacts with YB-1 and reduces its expression, which helps to restrain the progression of TNBC. Proteome profiling analysis indicates that the inhibition of YB-1 by SU056 can alter the proteins that regulate protein translation, an essential process for cancer cell growth. Preclinical studies on human cells, mice, and patient-derived xenograft tumor models show the effectiveness of SU056. Moreover, toxicological studies have shown that SU056 treatment and dosing are well tolerated without any adverse effects. Overall, our study provides a strong foundation for the further development of SU056 as a potential treatment option for patients with TNBC by targeting YB-1.

## Highlights

• SU056 inhibits triple-negative breast cancer growth in preclinical models
• SU056 targets Y-box binding protein-1 (YB-1)
• SU056 inhibits protein translation machinery through targeting YB-1

## Graphical abstract

**Figure:**
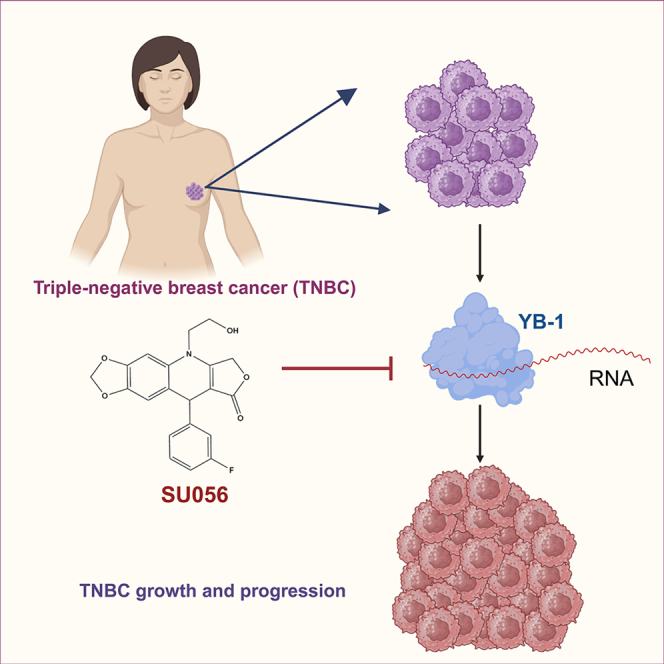

## Teaser

Dheeraj et al. identify SU056 as a potential therapeutic strategy for the treatment of triple-negative breast cancer through YB-1 inhibition. The inhibition of YB-1 results in a decrease in the expression of translational machinery proteins, leading to growth inhibition. SU056 shows promise for future therapeutic advancements.

## Introduction

Triple-negative breast cancer (TNBC) is a highly heterogeneous disease with distinct features at the pathological, molecular, and clinical levels that are associated with lower survival rates than other subtypes of BC.^1^ Approximately 12% of women within the US will be diagnosed with BC over the course of their lifetimes. TNBC is a highly aggressive subtype of BC and represents 15%–20% of all BC cases.^2^ TNBCs present a myriad of clinical challenges due to their heterogeneity, poor prognosis, high recurrence, and high incidence of metastasis.^3^

Y-box binding protein-1 (YB-1) is a well-established pro-oncogenic protein that belongs to the superfamily of highly conserved cold-shock proteins. Via its RNA-binding properties, YB-1 regulates different cellular processes including mRNA transcription, DNA repair, RNA splicing, transcript stability, and regulation of translation under physiological and pathological conditions.^4^^,^^5^ YB-1 regulates the mRNA stability, transcription, and translation of a wide range of genes implicated in cancer, including c-Myc, CD44, MET, HIF1α, etc.^6^^,^^7^^,^^8^^,^^9^^,^^10^ Overexpression of YB-1 is associated with tumor progression, cell invasion, and the development of treatment resistance in various cancers.^11^^,^^12^^,^^13^^,^^14^^,^^15^ YB-1 is associated with drug resistance through increased cancer cell stemness, cell proliferation, and drug export via transmembrane protein P-glycoprotein ATP-dependent efflux pump ABCB1 (MDR1).^16^^,^^17^^,^^18^^,^^19^ YB-1 is also involved in translational machinery, where it affects polysome assembly and regulates the translation of oncogenic mRNAs.^20^ The eukaryotic ribosome (80S) is a macromolecular complex composed of a large subunit (60S) and a small subunit (40S). The large subunit consists of 5S, 5.8S, and 28S rRNA and 47 ribosomal proteins (ribosomal proteins of large subunit [RPL]), while the small subunit comprises 18S rRNA and 33 ribosomal proteins (ribosomal proteins of small subunit [RPS]). The primary function of ribosomes is to translate genetic information into protein sequences; however, ribosomal proteins are also involved in extra-ribosomal functions such as proliferation, apoptosis, and DNA repair.^21^^,^^22^ The aberrant expression of different RP genes relates to tumorigenesis, which is sustained by increased translational levels across cancers. RPL15 (eL15) and RPL35 (uL29) expression in circulating breast tumor cells is correlated with increased proliferation and reduced apoptosis, as well as higher metastatic capacity.^23^ RPS15A was reported to promote proliferation and migration via the Akt pathway in glioblastoma.^24^ RPL32 was shown to have higher expression in BC, and its knockdown inhibited migration and invasion.^25^ Ribosomal protein L19 was found to be abundant in BC patient samples with higher ERBB2 levels.^26^ RPS15A was found to be overexpressed in glioblastoma compared with normal tissue.^27^ RPL34 knockdown inhibits migration of glioma cells through inhibition of the JAK/STAT3 signaling pathway.^28^ RPL11 is highly expressed in non-small cell lung cancer, where it promotes cell proliferation and migration by regulating endoplasmic reticulum stress and autophagy.^29^ Nucleolar stress induced by RNA polymerase I inhibition led to localization of ribosomal protein from the nucleolus to the nucleoplasm, where RPL5 and RPL11 bind with MDM2 and therefore increase the stabilization of p53.^30^ Depletion of ribosomal proteins RPL5 and RPL11 strongly suppressed cell cycle progression via reduced ribosome content and translational capacity.^31^

In previous work, we reported the development of a fluorine-based derivative of podophyllotoxin, SU056. SU056 was identified as a highly effective YB-1 inhibitor in ovarian cancer, where it inhibited tumor progression and synergized with chemotherapy.^32^ In this study, we report that SU056 is also effective in TNBC, where it alters key oncogenic protein translation processes through YB-1 inhibition and significantly decreases tumor progression.

## Results

### SU056 inhibits the growth of TNBC cells

**Figure 1:**
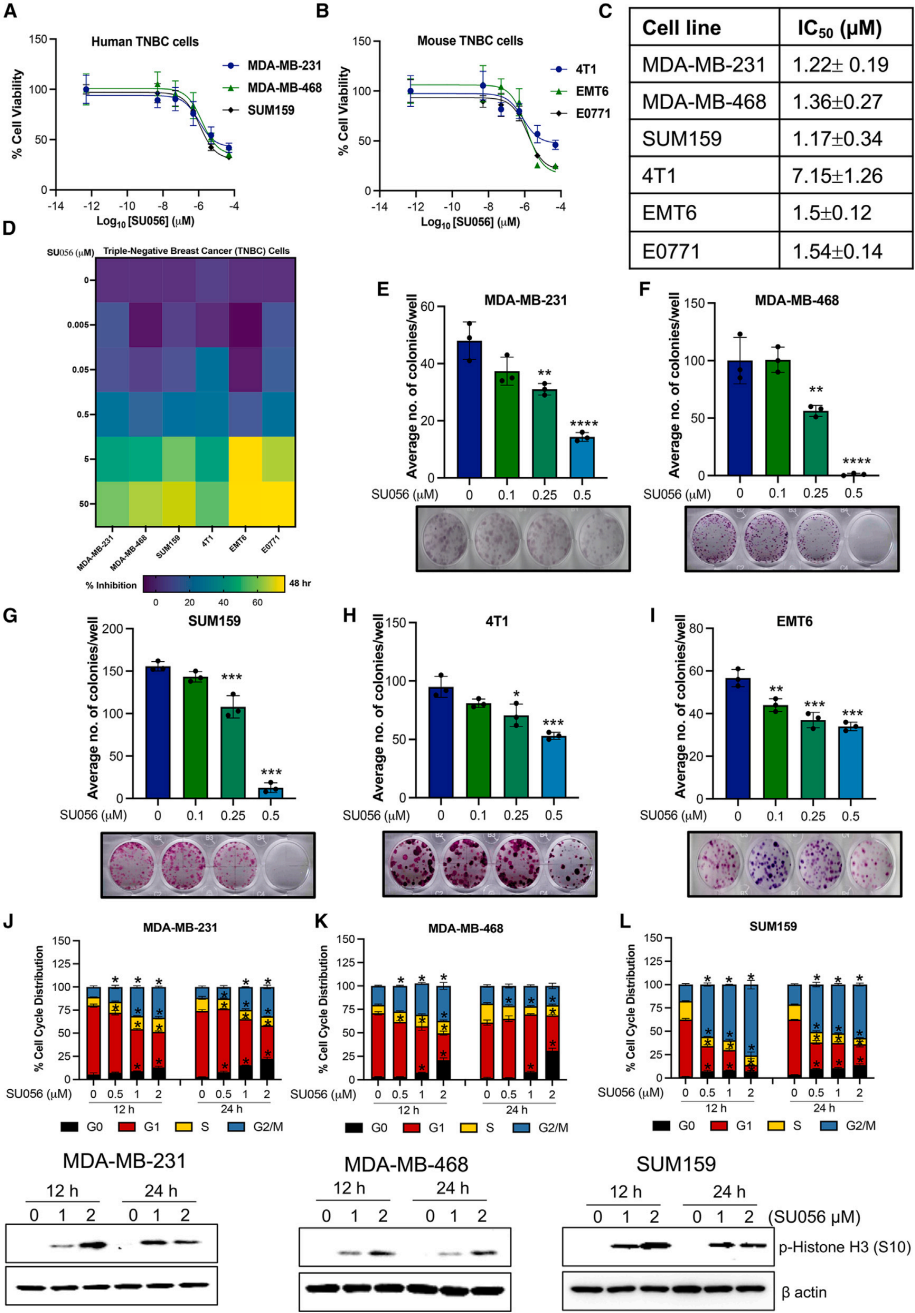
SU056 inhibits the growth of TNBC cells (A and B) The growth inhibitory effect of SU056 was evaluated using the MTT assay (0.5 mg/mL in 1× PBS). TNBC cell lines (MDA-MB-231, MDA-MB-468, SUM159, 4T1, EMT6, and E0771) were plated in a 96-well plate. The next day, cells were treated with vehicle (DMSO) alone or 0.005–50 μM SU056 in fresh medium. IC_50_ values were determined by MTT after 48 h. (C) Summary of IC_50_ values in micromolar concentrations in indicated TNBC cells. (D) Heatmap of growth inhibition of TNBC cell line panel upon treatment with SU056 for 48 h. Growth inhibition was measured by MTT assay. Growth inhibition is indicated in a gradient from blue (no growth inhibition) to yellow (growth inhibition). (E–I) Colony-formation assay of MDA-MB-231, MDA-MB-468, SUM159, 4T1, and EMT6 cells grown for 7–10 days in the presence of the indicated concentrations of SU056 or vehicle control. At the end of the treatment period, each well was stained with crystal violet, and colonies were counted under a 10× microscope. The representative wells are shown for each SU056 concentration. (J–L) Cell cycle analysis by flow cytometry using propidium iodide staining. MDA-MB-231, MDA-MB-468, and SUM159 cells were treated with vehicle control or the indicated concentration of SU056 for 12 and 24 h. At the end of treatment, cells were collected and analyzed using flow cytometry. SU056 treatment led to cell-cycle arrest at the G2/M phase in TNBC cells. The mitotic phase marker (histone H3) was elevated in SU056-treated groups for 12 and 24 h, as analyzed by western blotting. Data are shown as mean ± SD of triplicate values. Error bars represent ± SD. ∗*p* < 0.05, ∗∗*p* < 0.01, ∗∗∗*p* < 0.001, and ∗∗∗∗*p* < 0.0001. Significant difference compared with respective controls by Student’s t test or one-way ANOVA followed by Dunnett’s test.

The effects of SU056 on cell growth rates were screened in TNBC (MDA-MB-231, MDA-MB-468, SUM159, 4T1, EMT6, and E0771) cell lines. Cells were treated with different concentrations (0.005–50 μM) of SU056 for 48 h. After treatment, cells were measured for percentage of cell viability via the MTT assay as described earlier. IC_50_ values were calculated for each cell line based on cell viability (Figures 1A and 1B). The IC_50_ values for MDA-MB-231, MDA-MB-468, SUM159, 4T1, and EMT6 cells were 1.27 ± 0.19, 1.36 ± 0.27, 1.17 ± 0.34, 7.15 ± 1.26, and 1.5 ± 0.12 μM, respectively (Figure 1C). IC_50_ values for normal breast epithelial cells MCF10A and MCF12A were 4.77 ± 1.61 and 4.65 ± 2.25 μM, respectively (Figure S1A). The growth inhibition results are also presented as a heatmap for TNBC cells after 48 h treatment (Figure 1D). Next, we evaluated the effect of SU056 on the clonogenic potential of TNBC cells. Here, we observed that SU056 treatments (0.1, 0.25, and 0.5 μM) significantly decreased the number of colonies formed compared to untreated controls. We observed strong suppression of clonogenic potential in all TNBC cell lines at the 0.5 μM dose (Figures 1E–1I). We also tested the effect of SU056 on cell cycle progression. SU056 (0.5, 1.0, and 2.0 μM) treatment for 12 and 24 h showed significant G2/M phase cell-cycle arrest in TNBC cells. SUM159 cells showed 50%–58% cells in G2/M phase in the treated group at 24 h compared to 20% in the control group. In MDA-MB-231 cells, SU056 treatment caused more accumulation of cells in the G2/M phase of cell cycle, accounting for 34% compared to 12% after 24 h. Consistent with other cell lines, MDA-MB-468 cells also showed G2/M phase arrest (Figures 1J–1L). Cells were further analyzed for phospho-histone H3 (Ser10) levels, a marker for the mitotic phase, showing that SU056 treatment arrests TNBC cells in the mitotic phase. We observed a moderate increase in the cell cycle regulatory proteins (p21 and p27) (Figure S1B). We also tested SU056 effects on cell cycle regulation by synchronizing the cells in G2/M phase using nocodazole (200 nM for 16 h) followed by SU056 treatment for 24 h. Cells accumulated in G2/M phase upon nocodazole treatment, which, combined with SU056 treatment, resulted in increased cell death in MDA-MB-231 and MDA-MB-468 (Figures S1C and S1D). The prolonged cell-cycle arrest in both cells resulted in apoptotic cell death rates of 45%–50% and 39%–50% in MDA-MB-231 and MDA-MB-468, respectively. This was further confirmed by western blot of pro-apoptotic Bax and anti-apoptotic BCL-2 proteins. SU056 treatment induced Bax expression and inhibited BCL-2 expression (Figures S1E and S1F). These results show that SU056 inhibits growth and cell cycle progression across multiple TNBC cell lines.

### SU056 reduces tumor growth of human and mouse TNBC xenografts

**Figure 2:**
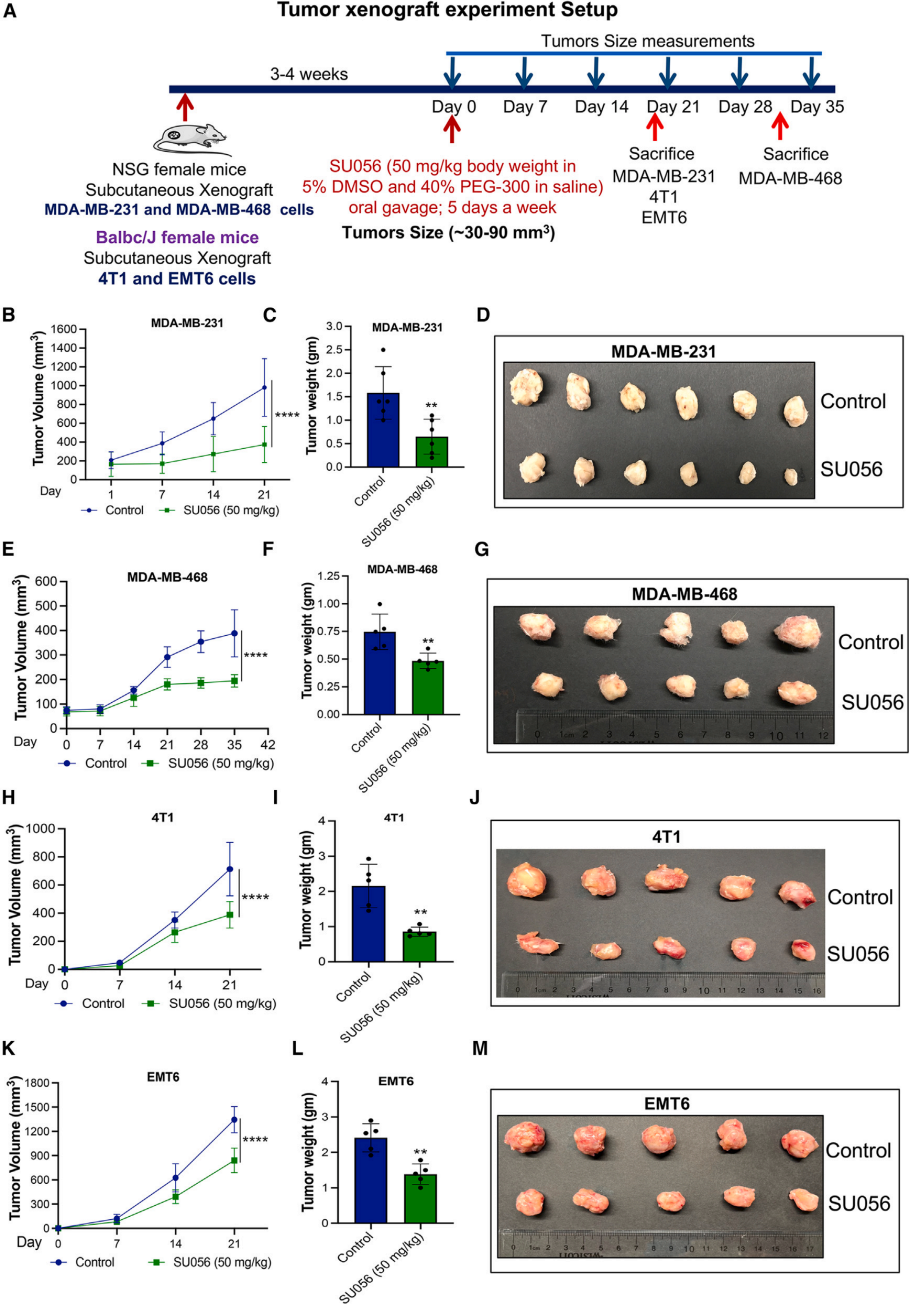
SU056 reduces tumor growth of human and mouse TNBC xenografts (A) Schematic of experimental setup for tumor xenograft study. Tumor xenografts were established in the right flank region of female NSG (MDA-MB-231 and MDA-MB-468) and Balbc/J (4T1 and EMT6) mice by implanting cells subcutaneously in 100 μL PBS. Treatment with SU056 (50 mg/kg) or vehicle control was started when the average tumor volume reached 30–80 mm^3^. Tumor volume and body weight were measured throughout the treatment period. (B) MDA-MB-231 tumor growth was graphed as a tumor volume as function of time. Animals were sacrificed when the average tumor volume of the vehicle control group reached ∼1,000 mm^3^. (C) At the end of the treatment period, tumors were collected and weighed. The bar graph represents the average tumor weight per group. (D) Tumors collected at the end of the indicated treatments. Representative image of tumors at the end of treatment period is shown. (E) MDA-MB-468 tumor volume and body weight were measured throughout the treatment period. Tumor growth was graphed as tumor volume through the treatment period. (F) At the end of treatment period, tumors were collected and weighed. The bar graph represents the average tumor weight per group. (G) Animals were sacrificed when the average tumor volume of vehicle control group reached 400 mm^3^. Tumors were collected at the end of indicated treatments. Representative image of tumors at the end of treatment period is shown. (H) 4T1 tumor xenograft growth was graphed as tumor volume as function of time. (I) At the end of treatment period, tumors were collected and weighed. The bar graph represents the average tumor weight per group. (J) Tumors collected at the end of indicated treatments. Representative image of tumors at the end of treatment period is shown. (K) EMT6 tumor xenograft growth was graphed as tumor volume throughout the treatment period. (L) At the end of treatment period, tumors were collected and weighed. The bar graph represents the average tumor weight per group. (M) Tumors collected at the end of the indicated treatments. Representative image of tumors at the end of the treatment period is shown. Data are shown as mean ± SD. Error bars represent ± SD. ∗*p* < 0.05, ∗∗*p* < 0.01, ∗∗∗*p* < 0.001, and ∗∗∗∗*p* < 0.0001. Significant difference compared with respective controls by Student’s t test or one-way ANOVA followed by Dunnett’s test.

Our results thus far suggest that SU056 reduces TNBC cell proliferation. To test this observation *in vivo*, we used xenografts of TNBC cell lines tested above to measure the effects of SU056. First, MDA-MB-231 (3 × 10^6^) and MDA-MB-468 (5 × 10^6^) were subcutaneously xenografted, and once tumors reached approximately 50–100 mm^3^, daily treatment with 50 mg/kg SU056 or vehicle control given via oral gavage was initiated (Figure 2A). In the MDA-MB-231 tumor xenograft, SU056 treatment arrested the tumor growth by 48% (*p* < 0.0001) compared to the vehicle control group (Figure 2B). SU056 treatment reduced tumor weight by 44.4% (*p* < 0.0068) as compared with vehicle control (Figures 2C and 2D). In a second model, MDA-MB-468, SU056 treatment reduced primary tumor growth by 49.8% (*p* < 0.0001) compared with vehicle control (Figure 2E). SU056 treatment reduced tumor weight by 35.1% (*p* < 0.0099) as compared with vehicle control (Figures 2F and 2G).

To build upon these observations, we also tested syngeneic mouse TNBC models. 4T1 (1 × 10^5^) and EMT6 (1 × 10^5^) syngeneic mouse cell lines were subcutaneously implanted as above, and SU056 treatment was initiated when tumors were ∼25–50 mm^3^. In the 4T1 model, SU056 treatment reduced the primary tumor load by 45.53% (*p* < 0.0001) and tumor weight by 61% (*p* < 0.0017) compared with vehicle control (Figures 2H–2J). In the EMT6 model, SU056 treatment decreased tumor growth by 37.47% (*p* < 0.0001) in comparison to the control group (Figure 2K) with 42.53% (*p* < 0.0016) reduction in tumor weight (Figures 2L and 2M). The body weight of animals remained the same throughout treatment (Figures S2A–S2D). Overall, these results suggest that SU056 exhibits therapeutic effects in tumor xenograft models.

### SU056 reduces the tumor growth of patient-derived TNBC xenografts

**Figure 3:**
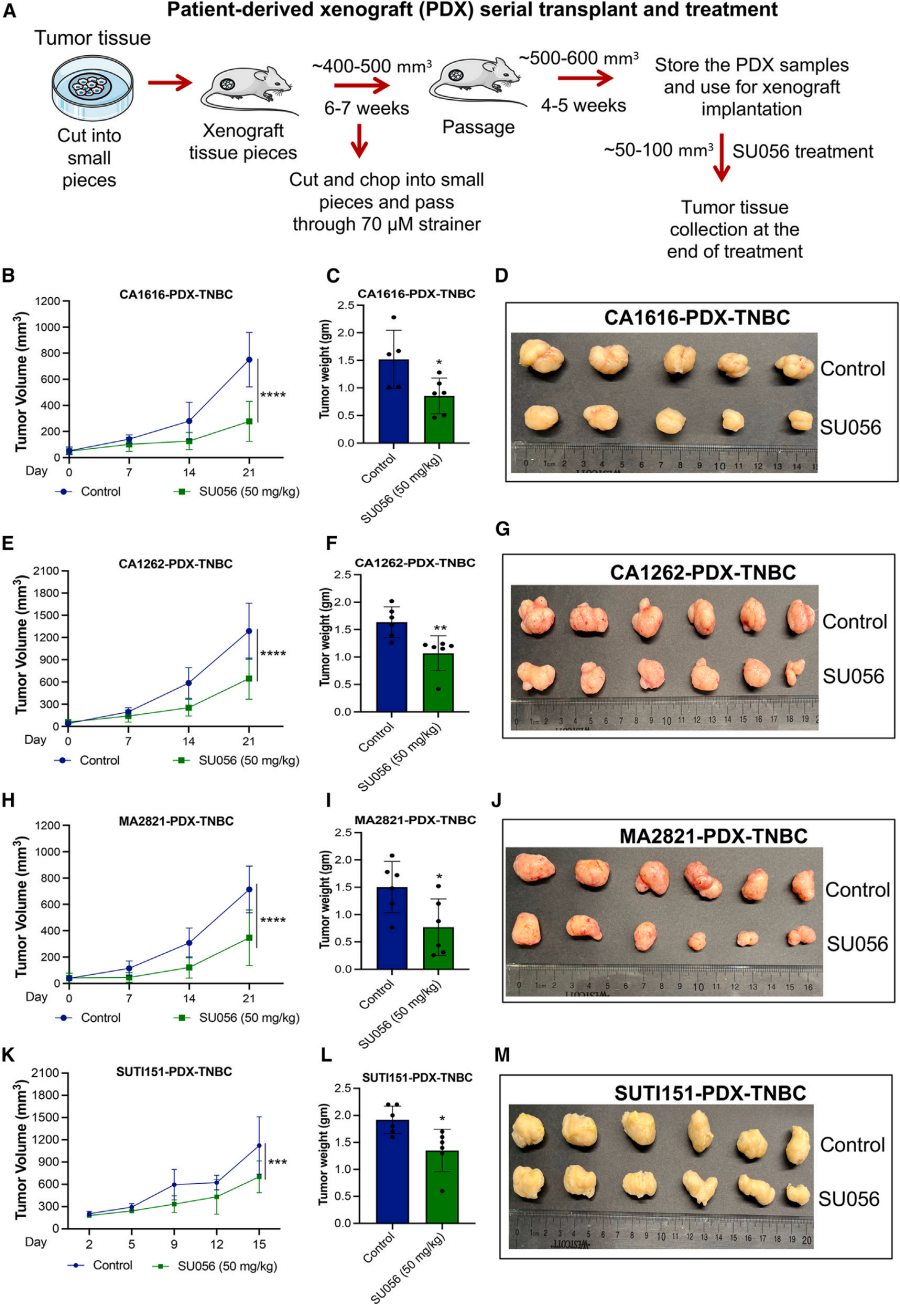
Effect of SU056 on the growth of PDX models of TNBC (A) Schematic diagram of PDX models. Patient tumor samples were received and finely chopped with a scalpel and passed through a syringe followed by straining through a 70 μM filter. Xenografts were generated by implanting cells mixed with Matrigel into the rear flank of female NSG mice. When the xenografts reached 400–500 mm^3^ tumor volume, animals were sacrificed, and tumors were collected. Tumors were serially passage into female NSG mice. These tumor samples were harvested, stored, and used for experiments. (B) CA1616-PDX-TNBC was implanted in the mammary fat pad in female NSG mice. Animals were randomized when tumor volume reached 100–200 mm^3^, and SU056 (50 mg/kg) or vehicle control treatment was started. Tumor volume was recorded as a function of time. (C) At the end of treatment period when vehicle control group tumors reached 1,000 mm^3^ size, tumors were collected and weighed. The bar graph represents the average tumor weight per group. (D) At the end of treatment period, tumors were collected. Representative image of tumors at the end of treatment is shown. (E) CA1262-PDX-TNBC was implanted in the mammary fat pad in female NSG mice. Animals were randomized when tumor volume reached 50 mm^3^ on average, and SU056 (50 mg/kg) or vehicle control treatment was started. Tumor volumes were recorded as a function of time. (F) At the end of treatment period when vehicle control group tumors reached 800 mm^3^ size, tumors were collected and weighed. The bar graph represents the average tumor weight per group. (G) At the end of treatment period, tumors were collected. Representative image of tumors at the end of treatment is shown. (H) MA2821-PDX-TNBc was implanted in the mammary fat pad in female NSG mice. Animals were randomized when tumor volume reached 50 mm^3^ on average, and SU056 (50 mg/kg) or vehicle control treatment was started. Tumor volumes were recorded as a function of time. (I) At the end of treatment period, tumors were collected and weighed. The bar graph represents the average tumor weight per group. (J) At the end of treatment when vehicle control group tumors reached 800 mm^3^ size, tumors were collected. Representative image of tumors at the end of treatment is shown. (K) SUTI151-PDX-TNBC was implanted in the mammary fat pad in female NSG mice. Animals were randomized when tumor volume reached 100–200 mm^3^, and SU056 (50 mg/kg) or vehicle control treatment was started. Tumor volume was recorded as a function of time. (L) At the end of treatment period when vehicle group tumors reached 1,000 mm^3^ size, tumors were collected and weighed. The bar graph represents the average tumor weight per group. (M) Tumors collected at the end of indicated treatments. Representative image of tumors at the end of treatment is shown. Data are shown as mean ± SD. Error bars represent ± SD. ∗*p* < 0.05, ∗∗*p* < 0.01, ∗∗∗*p* < 0.001, and ∗∗∗∗*p* < 0.0001. Significant difference compared with respective controls by Student’s t test or one-way ANOVA followed by Dunnett’s test.

The preclinical efficacy of SU056 was further tested in CA1616-patient-derived xenograft (PDX)-TNBC, CA1262-PDX-TNBC, MA2821-PDX-TNBC, and SUTI151-PDX-TNBC PDX models of TNBC (Figure 3A), showing 37%–63% tumor growth inhibition at doses of 50 mg/kg given orally over 21 days. In the PDX model CA1616-PDX-TNBC, SU056 treatment reduced tumor volume by 63.1% (*p* < 0.0001) compared to vehicle control (Figure 3B), and tumor weight was decreased by 43.6% (*p* < 0.0303) (Figures 3C and 3D). In the CA1262-PDX-TNBC model, SU056 treatment reduced tumor volume by 49.8% (*p* < 0.0001) compared to vehicle control (Figure 3E), and tumor weight was decreased by 34.67% (*p* < 0.0085) (Figures 3F and 3G). In another PDX model, MA2821-PDX-TNBC, SU056 treatment decreased tumor growth by 51.35% (*p* < 0.0001) (Figure 3H), and tumor weight was reduced by 48.89% (*p* < 0.0276) (Figures 3I and 3J). In the SUTI151-PDX-TNBC model, SU056 treatment reduced tumor volume by 37% (*p* < 0.0008) compared to the control group (Figure 3K), with a 29% (*p* < 0.0144) reduction in tumor weight (Figures 3L and 3M). The body weight of animals remained the same throughout treatment (Figures S2E–S2H). These results demonstrate SU056’s efficacy across a diverse range of TNBC models and show that it is an attractive therapeutic candidate. We also tested the effects of SU056 on metastasis in an orthotopic model of 4T1. The lung metastatic nodules were counted at the end of experiment, showing that the SU056-treated group had a 65.5% decrease in lung metastasis compared to the control group (Figures S2I–S2L). This result suggests that SU056 may also be a strong inhibitor of metastasis.

To evaluate SU056 as a potential therapeutic candidate, we evaluated the pharmacokinetics profile of SU056. Following oral administration of SU056 (20 mg/kg), plasma level peaked by 15 min (T_max_) with a half-life value of 60 min. A cytotoxic concentration of SU056 remained in the plasma for up to 6 h, where the maximum concentration (C_max_) reached 9.98 μg/mL (27.03 μM) (Figure S2M). In a liver microsome metabolism assay, we found that SU056 had a mean half-life of 40 min (Figure S2N). To test the maximum tolerated dose, both mice and rats were given SU056 at doses of 100, 200, and 400 mg/kg body weight via oral gavage over 72 h. The mice and rats were monitored for behavioral, neurological, and anatomical signs of toxicity and morbidity, and we found no observable changes in any of these metrics (Figures S3A–S3D). To test non-specific kinase inhibition, the kinase inhibition activity profile of SU056 (10 μM; 6-fold higher than IC_50_) was tested using an active site-directed competition binding assay (KINOMEscan, Eurofins Discovery). The results suggest that SU056 does not inhibit kinase activity (Figures S4A–S4C). An *in vitro* radioligand binding-and-enzyme and uptake assay suggested that SU056 does not affect major pharmacological parameters as tested by binding and enzyme assays, although SU056 also inhibits COX2 and PDE4D2 enzyme activity (Figures S4D and S4E). Overall, SU056 has a promising profile for a therapeutic drug.

### Identification of the protein translational process as a target of SU056

**Figure 4:**
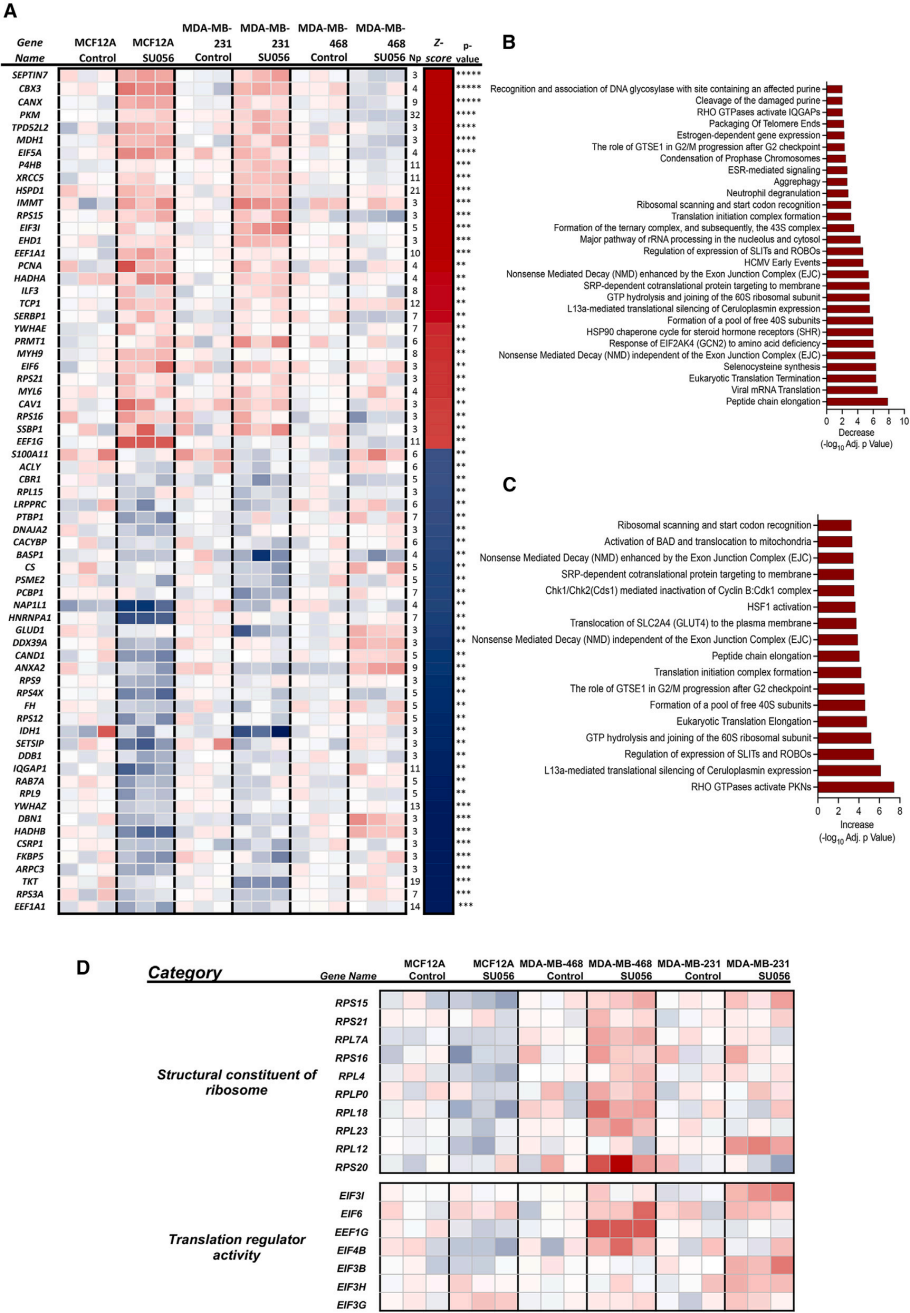
KEGG pathway analysis of proteome dataset (A and B) MDA-MB-231, MDA-MB-468, and MCF12A cells were treated with SU056 (1 μM) or vehicle control for 12 h. Cells were harvested, lysed, and digested with trypsin. Liquid chromatography-tandem mass spectrometry (LC-MS/MS) was performed. Gene set enrichment analysis was performed on the proteomic results to determine the enrichment of Kyoto Encyclopedia of Genes and Genomes pathways upon treatment with SU056. (A) Heatmap representation of modulated molecules with SU056 treatment. (B and C) Graph represents the (B) decrease and (C) increase of protein-associated biological processes upon SU056 treatment in cells. (D) Enrichment in pathways associated with structural constituents of the ribosome and translation regulator activity upon SU056 treatment.

To understand the effects of SU056 (1 μM, 12 h) on TNBC and normal cells, we performed a proteomic analysis to determine the biological pathways altered by treatment of TNBC cells with SU056. Protein translation processes, such as peptide chain elongation, eukaryotic translation termination, structural constituents of ribosomes, translation regulators, etc., were found to be modulated by SU056 treatment in TNBC cells compared to vehicle control (Figures 4A–4C). The affected proteins were primarily ribosome complex constituents including translation initiation factors and RPL and RPS subunit proteins (Figure 4D; Tables S1 and S2). These results suggest that protein translation pathways are affected by SU056 in TNBC cells.

**Figure 5:**
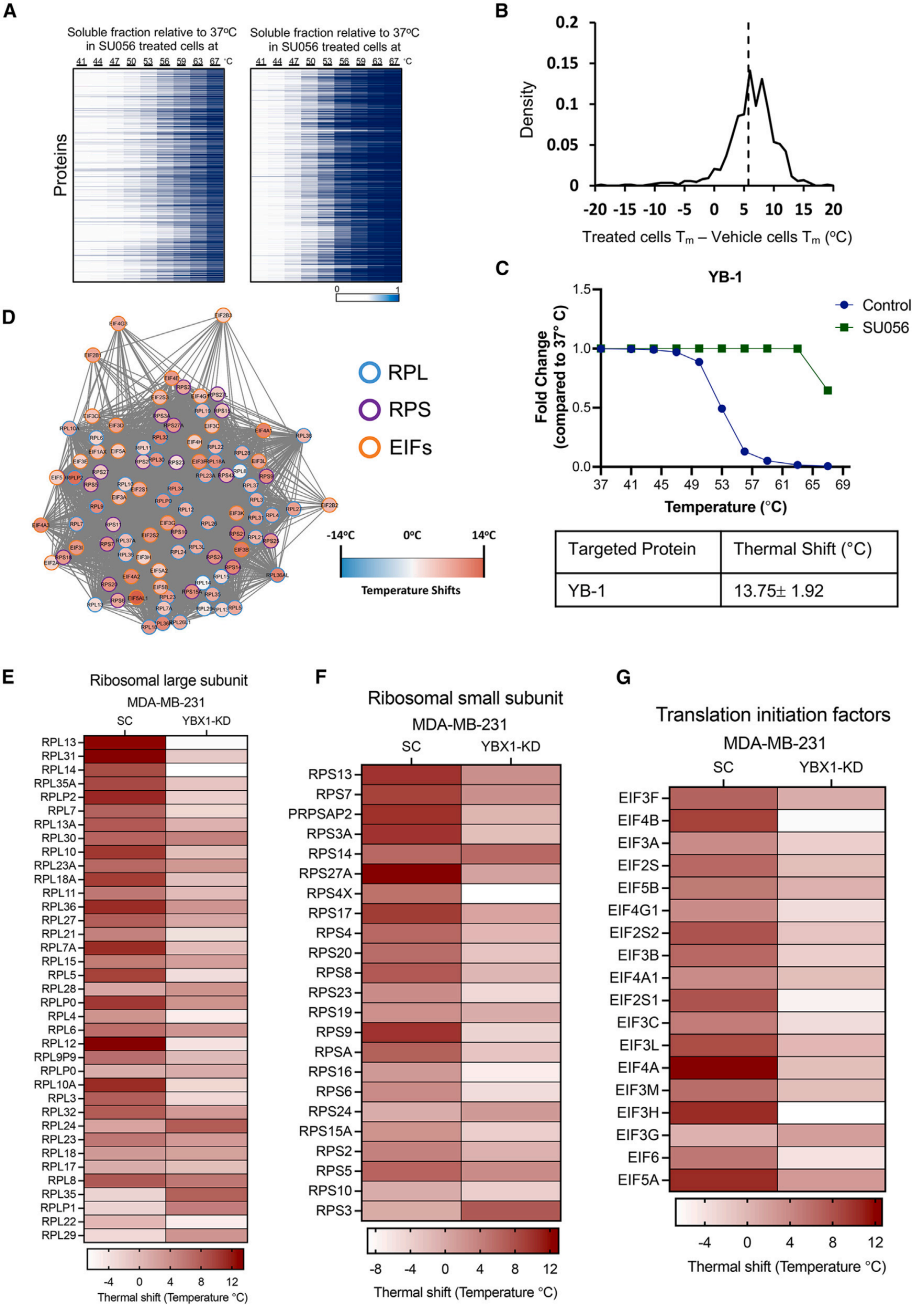
Target and pathway identification using cellular thermal shift assay Cellular thermal shift profiling of MDA-MB-231 cells for target identification of SU056. MDA-MB-231 cells were collected following 1.5 h treatment with SU056 (1 μM) or vehicle control. 1 × 10^6^ cells were aliquoted in PCR tubes and incubated at ten increasing temperatures (37°C, 41°C, 44°C, 47°C, 50°C, 53°C, 56°C, 59°C, 63°C, 67°C) for 3 min followed by 2 min incubation at room temperature. Cells were snap frozen and lysed by freeze-thaw cycling. Equal amounts of soluble proteins were labeled with tandem mass tag and run in LC-MS/MS analysis. (A) Heatmap representation of the thermal stability of soluble proteins in SU056- (left) or vehicle (right)-treated MDA-MB-231 cells. The soluble fraction of proteins (unbound proteins) compared to 37°C proteins was mapped as percentages (0–1) for both vehicle- and SU056-treated cells. (B) Density distributions of T_m_ shifts between SU056 and vehicle treatment. (C) The melting curve for YB-1 indicates a 13.75°C shift in melting temperature in the presence of SU056. (D) A StringDB plot of translation machinery proteins based on their temperature shifts between vehicle- and SU056-treated cells. eIF, eukaryotic initiation factors; RPL, ribosomal proteins of large subunit; RPS, ribosomal proteins of small subunit. (E–G) Heat of thermal shifts of RPLs, RPSs, and eIFs in MDA-MB-231-YBX1 knockdown (KD) conditions upon SU056 treatment.

To identify and better understand the target of SU056 in TNBC cells, we performed a cellular thermal shift assay (CETSA), in which protein-ligand interaction-mediated shifts in thermal stability curves of proteins are quantified in a global manner to identify the protein targets of small molecules. MDA-MB-231 cells were treated with SU056 at 1 μM or vehicle for 1.5 h followed by TMT labeling and liquid chromatography-tandem mass spectrometry analyses. The heatmap representing the thermal stability of soluble proteins compared to 37°C vehicle- or SU056-treated groups is shown in Figure 5A and Table S3. The density of protein melting temperatures, T_m_, was shifted to the right by SU056 treatment, reflecting higher temperatures of protein denaturation (Figure 5B). Previously, in our study of SU056 in an ovarian cancer model, we found that YB-1 is the target of this drug.^32^ SU056 treatment resulted in increasing the thermal stability of YB-1 by 13.75°C ± 1.92°C in MDA-MB-231 cells (Figure 5C). Comparing the thermal stability of the proteome from vehicle- and SU056-treated samples suggested that SU056 treatment increased the overall stability of translation-associated proteins, including eukaryotic initiation factors (eIFs) and RPL and RPS subunit proteins (Figure 5D), showing that protein translation pathways are modulated by SU056 in TNBC cells. Next, we tested the effect of SU056 on MDA-MB-231-YB-1 knockdown cells by CETSA analysis. We found that SU056 treatment does not increase the thermal stability of translation machinery molecules (RPLs, RPSs, and eIFs) in the YB-1 knockdown conditions as compared to scrambled control cells. In fact, most of the observed proteins of translation machinery showed a decrease in thermal stability, which suggests that YB-1 is a key molecule to mediate the protein translation process through ribosomal proteins (Figures 5E–5G; Table S4). In our results, SU056 modulates the protein translation process by targeting YB-1 protein and inhibiting TNBC progression.

### SU056 targets the translation-associated molecules in TNBC cells

**Figure 6:**
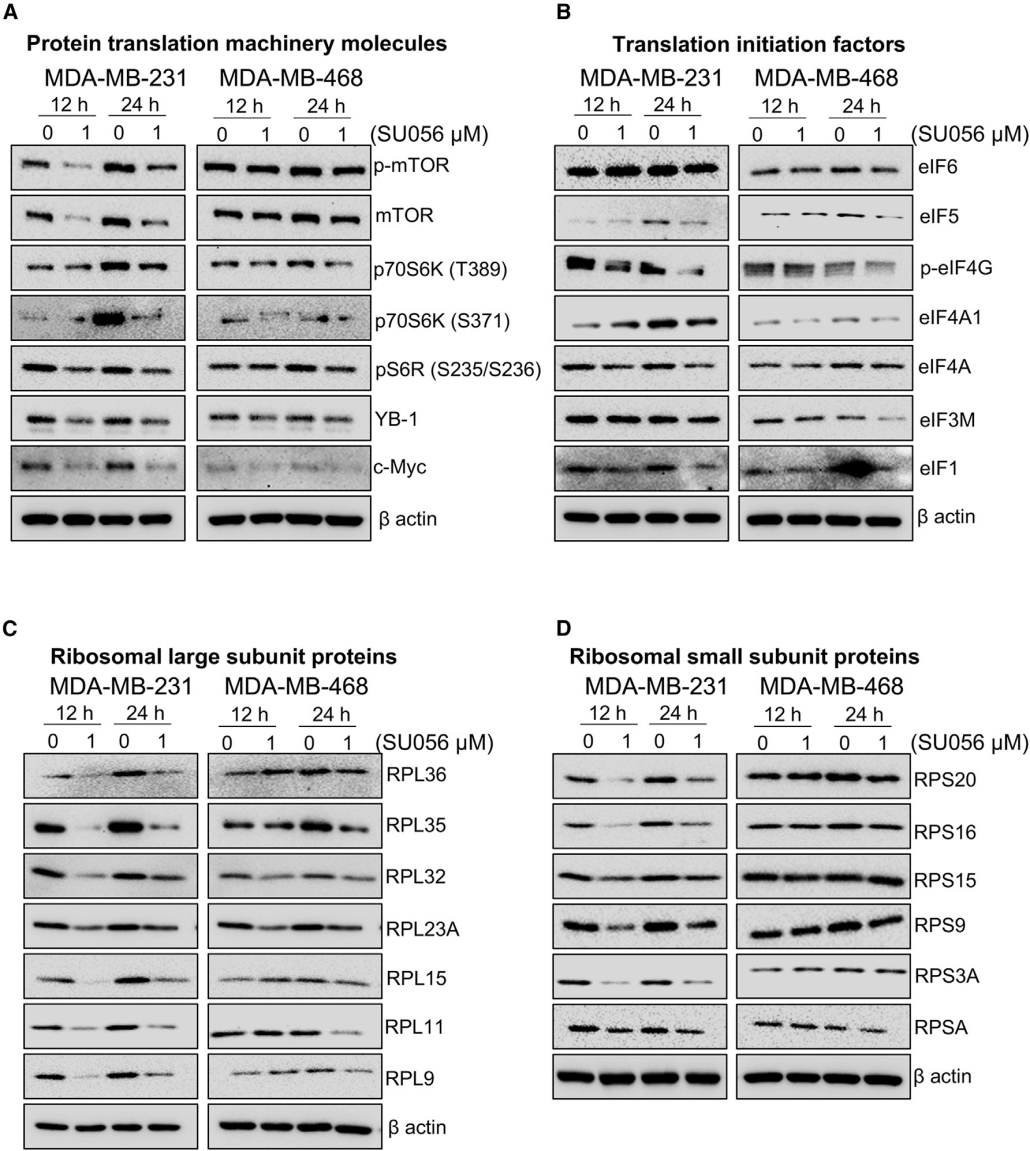
SU056 targets translation processes in TNBC cells MDA-MB-231 and MDA-MB-468 cells were treated for 12 and 24 h and total cell lysates were prepared. 10–20 μg protein was run on SDS-PAGE, and western blot analyses were performed for translation-associated molecules. β-Actin was probed to ensure equal protein loading. SU056 treatment inhibited protein translation-associated molecules in all TNBC models. (A) Protein translation machinery molecules. (B) Translation initiation factor molecules. (C) Ribosomal large subunit (RPL) proteins. (D) Ribosomal small subunit (RPS) proteins.

Since protein translation was identified as a target of SU056, we analyzed multiple mTORC1-related signaling proteins commonly dysregulated in cancer. SU056 inhibited the phosphorylation of mTOR (S2448) and decreased overall levels of mTOR across the TNBC (MDA-MB-231, MDA-MB-468, and SUM159) cell lines. The downstream effectors of mTOR signaling, p70 S6 kinase and S6 ribosomal (S6R) protein, which regulate cell growth, were also modulated by SU056. SU056 decreased the phosphorylation of p70S6 kinase (T389 and S371) and S6R (S235/S236). These are important components of the mTOR signaling complex showing that SU056 affects cell growth regulation. In our earlier report on SU056 in ovarian cancer, YB-1 was identified as a target of SU056. Here, we measured the levels of YB-1 with SU056 treatment in TNBC cells and found that SU056 treatment decreased YB-1 expression in TNBC cells alongside c-Myc, an oncogenic transcription factor (Figures 6A and S5A). SU056 treatment also decreased the levels of various translation initiation factors in TNBC cells, including eIF4F complex molecules, eIF4G (a scaffold molecule), and eIF4A (an RNA helicase), which have been reported to interact with YB-1.^33^ eIF1, eIF3, and eIF5 are also part of the preinitiation complex (PIC), where they assist in start codon scanning (Figures 6B and S5B). These proteins are also downregulated by SU056 treatment, which profoundly perturbed protein translation.

The translation process is initiated upon the assembly of the 40S and 60S ribosomal subunits at the start codon. Here, we tested the effects of SU056 on the expression of different ribosomal proteins of both the small (RPS) and large (RPL) subunits. SU056 treatment led to lower expression of both RPSs and RPLs. Numerous RPLs including RPL36, RPL35, RPL32, RPL23A, RPL15, RPL11, and RPL9 were decreased upon SU056 treatment for 12 and 24 h in TNBC cells (Figures 6C and S5C). This effect extended to different RPS molecules including RPS20, RPS16, RPS15, RPS9, RPS3A, and RPSA. SU056-mediated effects were strongest in MDA-MB-231 cells, and in MDA-MB-468 and SUM159 cell lines, SU056 exhibited only moderate inhibition across the treatment time points (12 and 24 h) (Figures 6D and S5D). To further understand whether this is a stress response, we checked the expression of key integrated stress response regulators PERK, eIF2α, and ATF4. SU056 treatment did not induce integrated stress response activation, as we did not see overall changes of its key factors (Figure S5E). The decrease in different ribosomal proteins suggests that SU056 acts to combat TNBC progression via modulation of protein translation, suggesting a potential mechanism for cancer treatment.

### SU056 interacts with YB-1

**Figure 7:**
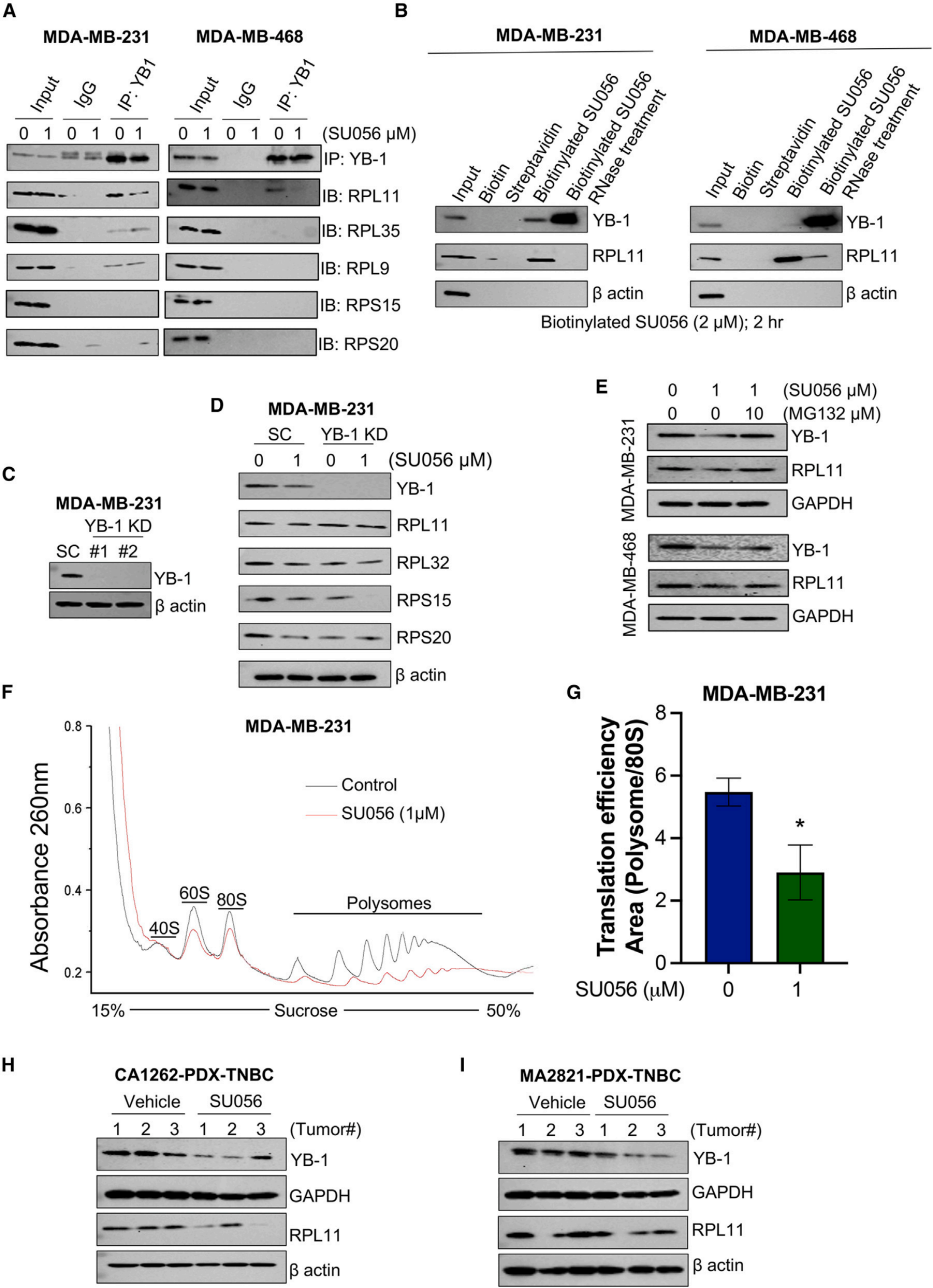
SU056 interacts with YB-1 (A) Immunoprecipitation of YB-1 from protein lysates of SU056-treated MDA-MB-231 and MDA-MB-468 cells using YB-1 antibody and control rabbit immunoglobulin G (IgG) antibody. Samples were processed and 10–20 μg protein was run on SDS-PAGE, and western blot were performed for translation-associated molecules (RPLs and RPSs). (B) Pull down with biotinylated SU056 followed by western blot for YB-1 and RPL11. β-Actin acted as a control following treatment (2 h) of MDA-MB-231 and MDA-MB-468 cells with SU056 (2 μM). (C) Western blots for YB-1 and actin on cell lysates from MDA-MB-231 cells expressing control short hairpin RNA (shRNA; SC) or shRNAs targeting YB-1 (YB-1 KD). (D) Western blots for YB-1, RPLs, RPSs, and actin on cell lysates from MDA-MB-231 cells expressing SC or YB-1 KD and treated with SU056 (1 μM for 24 h). (E) MDA-MB-231 and MDA-MB-468 cells were treated with vehicle, SU056 (1 μM), or MG132 (10 μM) + SU056 (1 μM). Cells were harvested after 15 h incubation and subjected to western blot for YB-1, RPL11, and GAPDH. (F and G) Line and bar graphs represent the change in translation efficiency upon SU056 treatment in MDA-MB-231 cells. (H and I) Protein lysates from tumor were processed for immunoblotting as described in the STAR Methods. 10 μg protein was run on SDS-PAGE, and western blot were performed for YB-1 and RPL11 using GAPDH and β-actin as a loading control. Data are shown as mean ± SD. Error bars represent ± SD. ∗*p* < 0.05, ∗∗*p* < 0.01, ∗∗∗*p* < 0.001, and ∗∗∗∗*p* < 0.0001. Significant difference compared with respective control by Student’s t test.

Our proteomic and CETSA datasets showed that RPL and RPS proteins are both enriched. Therefore, we performed immunoprecipitation to understand whether YB-1 interacts directly with RPL and RPS. The results suggest that YB-1 interacts with different RPL proteins, including RPL11. However, SU056 treatment inhibits this YB-1 and RPL11 interaction (Figures 7A and 7B). Next, we performed a pull-down assay with biotinylated SU056 (2 μM, 2 h) in MDA-MB-231 and MDA-MB-468 cell lines and found that it physically interacts with YB-1, leading to inhibition of the RPL11 interaction. Interestingly, we also found that whether YB-1 was in its RNA-bound or free state influenced its interaction with SU056. In RNase-treated protein lysates, the interaction between YB-1 and SU056 was increased by 3.13- and 18.76-fold in MDA-MB-231 and MDA-MB-468 cells, respectively. Moreover, pull down with biotinylated SU056 in the RNA-free condition suggests that the SU056 and RPL11 interaction is dependent on the presence of RNA. SU056 interacts with YB-1 in both the presence and absence of RNA; however, the interaction of SU056 with YB-1 is stronger when it is in an RNA-free state (Figure 7B). To functionally test YB-1 as a target of SU056, we generated MDA-MB-231 cells with knockdown of YB-1 using short hairpin RNA (Figure 7C). YB-1 knockdown led to a decrease in the response of SU056 on the expression level of RPL11, further supporting YB-1 as a target of SU056 that leads to inhibition of RPL11 in cells and inhibits protein translation (Figure 7D). Treatment with the proteasomal inhibitor MG132 partially reversed the SU056-induced decrease in levels of YB-1 and RPL11, suggesting that SU056 leads to degradation of YB-1 and RPL11 (Figure 7E). The polysome profiling analysis results suggest that SU056-treated cells have reduced translational efficiency compared to control cells (Figures 7F and 7G). Next, we tested the effect of SU056 on two PDX models of TNBC (CA1262-PDX-TNBC and MA2821-PDX-TNBC) for YB-1 and RPL11 expression. Daily oral SU056 (50 mg/kg) treatment for 3 weeks resulted in a decrease in the levels of YB-1 and RPL-11 in tumors (Figures 7H and 7J). Overall, the data suggest that SU056 targets YB-1 and in turn inhibits the translation process, blocking TNBC from using increased translation to sustain tumor progression.

## Discussion

Our study provides insight into the regulation of YB-1 by SU056 in TNBC. YB-1 functions as a transcription factor that regulates the transcription and translation of genes involved in proliferation, apoptosis, stress response, DNA repair, and drug resistance.^34^ It is well established that ribosomal proteins are not only limited to ribosome complex and protein biosynthesis but are also involved in the development of cancer.^35^^,^^36^ The continued progression of treated cancers demands increased protein synthesis capacity, both to sustain cell proliferation and to counteract the effects of ongoing treatments. This study shows that SU056 disrupt protein translation machinery by targeting YB-1, resulting in potent anti-cancer effects in TNBC models. Initial tests of SU056 in TNBC cell lines showed significant induction of G2/M cell-cycle arrest accompanied by loss of cell viability and clonogenic potential with increased cell death. Analysis of SU056-treated TNBC lysates via proteomics and KEGG pathway analysis revealed a significant alteration of multiple ribosome-associated pathways, including peptide chain elongation, mRNA translation, translational silencing, and transcript scanning.

Protein translation is a universal process needed for cellular homeostasis. Mitogenic signals activate Akt, which phosphorylates and activates mTOR, a Ser/Thr protein kinase. Activated mTOR regulates key aspects of cell proliferation, such as production of protein, nucleotides, and lipids, while suppressing autophagy.^37^ SU056 treatment decreased mTOR phosphorylation and thus reduced protein synthesis. This effect was also reflected in decreased phosphorylation of related factors p70S6 kinase (S6K1) and ribosomal protein S6 at key sites. SU056-treated S6K1 exhibited reduced phosphorylation at the Thr389 site, which is phosphorylated by activated mTORC1, as well as at the S371 site, itself phosphorylated by glycogen synthase kinase 3 β associated with Wnt/β-catenin and epithelial-to-mesenchymal transition signaling in TNBC after mTORC1 de-repression via PP2A. This effect may be cooperative, as S371 phosphorylation increases subsequent Thr389 phosphorylation, which in turn drives ribosome biogenesis.^38^^,^^39^ SU056 treatment also decreased the phosphorylation of ribosomal protein S6, a downstream effector of activated S6K1, at the S240 and S244 sites, which are required for its cap-binding activity in translation initiation.^40^ Decreased phosphorylation of mTOR and its downstream substrates reduces protein synthesis in cancer cells.

The translation initiation process is the rate-limiting step in protein synthesis and is coordinated by the activity of multiple eIFs. Here, we find that SU056 alters multiple components of the translation initiation complex by reducing the levels of several eIFs. Components of the 43S PIC and the eIF4F complex, which binds the m^7^G-5′ cap of mRNAs to stabilize RNAs for interaction with the components of the 43S PIC and subsequent assembly of the full 80S ribosome, were both affected by SU056 treatment. Sharp changes in the ratio of ribosomes to mRNAs can modify overall protein synthesis, leading to deleterious effects on cells.^41^^,^^42^ However, in line with a mechanism that broadly affects ribosome regulation, we also found that SU056 treatment decreased the levels of myriad ribosomal proteins, including large subunit proteins RPL9, RPL11, RPL15, RPL23A, RPL32, RPL35, and RPL36 as well as small subunit proteins RSPA, RPS2A, RPS9, RPS15, RPS16, and RPS20. With these observations, it is evident that SU056 acts against TNBC progression through inhibition of ribosomal proteins.

Whether this effect is restricted to canonical ribosome assembly or YB-1-promoted assembly of alternate ribosome complexes on stress-associated RNA internal ribosomal entry sites is unknown. Notably, consistent with our previous study in ovarian cancer, SU056 treatment also inhibited YB-1 and downstream c-Myc activity, which drives increased translation activity and proliferation. YB-1 regulates c-Myc expression in various models through its binding to the Myc transcript and helps its recruitment to polysomal chains.^10^ YB-1 is a stress-responsive RNA-binding protein involved in mRNA stability and ribosome assembly whose overexpression is associated with increased cancer progression and chemoresistance. It has been reported that YB-1 is an oncogene in BC, where it promotes proliferation, metastasis, and drug resistance.^19^^,^^43^^,^^44^ The exact mechanism by which YB-1 bridges mRNA stability and protein translation processes is currently unclear. Through CETSA analysis, we found that YB-1 knockdown reverses the effect of SU056 on the thermal stability of ribosomal proteins, which suggests that YB-1 is a critical player in the translational impact of SU056. In this study, we found that YB-1 interacts with RPL11. The interaction between YB-1 and RPL11 may have a role in the regulation of overall translation activity, with loss of RPL11 leading to reduced ribosome content and protein synthesis that halts cell cycle progression.^31^ YB-1 autoregulates its own translation and maintains high expression with active mTOR signaling.^45^^,^^46^ The interaction between SU056 and YB-1 is also influenced by the RNA-bound state of YB-1, as observed in RNase-treated protein lysates, where SU056 binds YB-1 more strongly in the absence of RNAs. This suggests that SU056 interacts with YB-1 on its RNA-binding sites. In a structure-based pharmacophore model, SU056 demonstrates binding with YB-1 through π-π stacking or π-cation interactions, mimicking the protein-RNA interactions as the CSD.^47^ Our findings show that SU056 inhibits YB-1 binding to nascent mRNAs via its RNA-binding site and also inhibits the interaction between YB-1 and RPL11, in turn suppressing translation. YB-1 knockdown reversed SU056-mediated translation inhibition, suggesting that this mechanism depends on YB-1. The translational efficiency was also decreased by SU056.

Based on the above *in vitro* results from several TNBC cell lines, we also investigated the therapeutic potential of SU056 in cell line xenografts and found broad and potent anti-cancer activity across a wide variety of models. These data show that SU056 significantly restrained the growth of multiple TNBC models, including in the overly aggressive and treatment-resistant syngeneic 4T1 model. The growth inhibition efficacy of SU056 was consistent across human and mouse xenografts. The delayed growth effect was accompanied by reduced metastatic potential. Measurement of mouse body weight and a maximum tolerated dose study in mouse and rat showed that SU056 treatment is very well tolerated even at doses 8× those used for treatment studies, with a promising pharmacokinetic profile for additional translational investigation. These data show that attenuation of TNBC protein synthesis through YB-1 inhibition using SU056 is a promising strategy for the treatment of TNBC.

### Limitation of the study

Further studies on various cancer histologies are necessary to expand the understanding of YB-1 inhibition by SU056 beyond TNBC. While this manuscript demonstrates a link between YB-1 and translational machinery, further investigation is needed to determine the interplay between translational machinery molecules and YB-1 in cancer.

## STAR★Methods

### Key resources table

**Table:** 

REAGENT or RESOURCE	SOURCE	IDENTIFIER
**Antibodies**		

pHistone H3	Cell Signaling Technology	Cat#3377; RRID: AB_1549592
β actin	Cell Signaling Technology	Cat#4970S; RRID: AB_2223172
YB-1	Cell Signaling Technology	Cat#4202S; RRID: AB_1950384
pmTOR	Cell Signaling Technology	Cat#5536T; RRID: AB_10691552
mTOR	Cell Signaling Technology	Cat#2983S; RRID: AB_2105622
p70S6K (T389)	Cell Signaling Technology	Cat#9234T; RRID: AB_2269803
p70S6K (S371)	Cell Signaling Technology	Cat#9208T; RRID: AB_330990
pS6R (S235/S236)	Cell Signaling Technology	Cat#2211S; RRID: AB_331679
c-myc	Novus Biological	Cat#NB600-302SS; RRID: AB_2037063
eIF6	Cell Signaling Technology	Cat#3263S; RRID: AB_2293295
eIF5	Thermo Scientific	Cat#PA5-51472; RRID: AB_2640977
peIF4G	Cell Signaling Technology	Cat#2441T; RRID: AB_2277632
eIF4A1	Cell Signaling Technology	Cat#2490; RRID: AB_823487
eIF4A	Cell Signaling Technology	Cat#2013; RRID: AB_2097363
eIF3M	Novus Biological	Cat#NBP1-56654; RRID: AB_11030684
eIF1	Cell Signaling Technology	Cat#12496S; RRID: AB_2721252
RPL36	Thermo Scientific	Cat#PA5-61449; RRID: AB_2646745
RPL35	Thermo Scientific	Cat#PA5-52245; RRID: AB_2646744
RPL32	Thermo Scientific	Cat#PA5-88906; RRID: AB_2805210
RPL23A	Abcam	Cat#ab157110; RRID: N/A
RPL15	Abcam	Cat#ab155802; RRID: N/A
RPL11	Thermo Scientific	Cat#37–3000; RRID: AB_2533310
RPL9	Novus Biological	Cat#NBP2-20219; RRID: N/A
RPS20	Novus Biological	Cat#NBP1-80804; RRID: AB_11017106
RPS16	Novus Biological	Cat# NBP1-80025; RRID: AB_11002635
RPS15	Thermo Scientific	Cat#PA5-62977; RRID: AB_2646776
RPS9	Novus Biological	Cat#NBP2-22298; RRID: N/A
RPS3A	Novus Biological	Cat#NBP2-20225; RRID: N/A
RPSA	Thermo Scientific	Cat#PA5-27281; RRID: AB_2544757
p21	Cell Signaling Technology	Cat#2947T; RRID: AB_823586
p27	Cell Signaling Technology	Cat#3686T; RRID: AB_2077850
Bax	Cell Signaling Technology	Cat#5023S; RRID: AB_10557411
BCL2	Cell Signaling Technology	Cat#2876S; RRID: AB_2064177
PERK	Cell Signaling Technology	Cat#3192S; RRID: AB_2095847
peIF2***α***	Cell Signaling Technology	Cat#3398S; RRID: AB_2096481
eIF2***α***	Cell Signaling Technology	Cat#5324S; RRID: AB_10692650
ATF4	Cell Signaling Technology	Cat#11815S; RRID: AB_2616025
Anti-mouse IgG HRP-linked antibody	Cell Signaling Technology	Cat#7076; RRID: AB_330924
Anti-rabbit IgG HRP-linked antibody	Cell Signaling Technology	Cat#7074; RRID: AB_2099233

**Biological samples**		

MA2821-PDX-TNBC	PDMR NCI Patient-Derived Models Repository	Specimen ID; 021-R RRID: N/A
CA1616-PDX-TNBC	PDMR NCI Patient-Derived Models Repository	Specimen ID; 120-R RRID: N/A
CA1262-PDX-TNBC	PDMR NCI Patient-Derived Models Repository	Specimen ID; 296-R RRID: N/A
SUT151-PDX-TNBC	Zhang et al.^48^	RRID: N/A

**Chemicals, peptides, and recombinant proteins**		

RPMI-1640	Corning	Cat#10-040-CV
DMEM	Corning	Cat#10-013-CV
MEGM media	Lonza	Cat#CC-3150
Waymouth MB 752/1	Sigma Aldrich	Cat#W1625
FBS	Corning	Cat#35-015-CV
Antibiotic-Antimycotic solution	Gibco	Cat#15240062
M-PER™ lysis solution	Thermo Scientific	Cat#78503
Halt protease and phosphatase inhibitor cocktail	Thermo Scientific	Cat#78440
Magnetic conjugate streptavidin bead	CST	Cat#5947
Biotin	Sigma Aldrich	Cat# B4639
MG132	SelleckChem	Cat#S2619

**Critical commercial assays**		

Tandem Mass Tag (TMT)	Thermo Scientific	Cat#90110, Cat#90061
KINOMEscan™ Profiling	Eurofins Discovery	scanMAX
*In Vitro* Pharmacology	Eurofins Discovery	N/A
SelectScreen kinase profiling	Thermo Scientific	N/A

**Deposited data**		

The mass spectrometry proteomics data	PRIDE Archive	(http://www.ebi.ac.uk/pride/archive/) Identifier# PXD042380

**Experimental models: cell lines**		

MDA-MB-231	NCI cell line repository (DTP)	RRID: CVCL_0062
MDA-MB-468	NCI cell line repository (DTP)	RRID: CVCL_0419
SUM159	Canary Center at Stanford for Cancer Early Detection, Stanford University, Palo Alto, CA	RRID: CVCL_5423
4T1	ATCC	Cat#CRL-2539: RRID; CVCL_0125
EMT6	ATCC	Cat#CRL-2755; RRID: CVCL_1923
E0771	ATCC	Cat#CRL-3461; RRID: CVCL_GR23

**Experimental models: Organisms/strains**		

NOD.Cg-Prkdcscid Il2rgtm1Wjl/SzJ	Jackson laboratories	Strain #:005557
BALB/cJ	Jackson laboratories	Strain# BALB/cJ
Sprague-Dawley rat	Charles river laboratories	Strain#Crl:CD(SD)001; RRID: RGD_734476

**Recombinant DNA**		

pLV[shRNA]-EGFP:T2A:Puro-U6>hYBX1[shRNA#1] shRNA sequence: CCTGTTAATAAAGGTCTTAAA	VectorBuilder Inc	N/A
pLV[shRNA]-EGFP:T2A:Puro-U6>hYBX1[shRNA#2] shRNA sequence: CCAGTTCAAGGCAGTAAATAT	VectorBuilder Inc	N/A
pLV[shRNA]-EGFP:T2A:Puro-U6>Scramble[shRNA#1] shRNA sequence: CCTAAGGTTAAGTCGCCCTCG	VectorBuilder Inc	N/A

**Software and algorithms**		

GraphPad prism	GraphPad Software	https://www.graphpad.com/

### Resources availability

#### Lead contact

Any requests for resources and reagents or information should be directed to the lead contact, Sanjay V. Malhotra (malhotsa@ohsu.edu).

#### Materials availability

The materials generated in this study will be distributed upon request. There are restrictions to availability due to a Material Transfer Agreement (MTA).

#### Data and code availability

• Data: The mass spectrometry proteomics data have been deposited to the PRIDE Archive (http://www.ebi.ac.uk/pride/archive/) via the PRIDE partner repository with the dataset identifier PXD042380.
• Code: This study did not result in any development of original code.
• Any additional information required to reanalyze the data reported in this work paper is available from the lead contact upon request.

### Experimental model and study participant details

#### Cell lines

Human triple negative breast cancer cell lines MDA-MB-231, MDA-MB-468, and SUM159 (ATCC, USA) were cultured in DMEM with 10% FBS (Corning, USA; #35-015-CV), 1% sodium pyruvate, and 1% penicillin/streptomycin at 37°C in a humidified 5% CO_2_ incubator. 4T1 and EMT6 mouse syngeneic cell lines were obtained from ATCC and maintained in RPMI-1640 and Waymouth’s MB 752/1 Medium with 15% FBS and supplemented with 1% penicillin/streptomycin, respectively. Normal breast epithelial cells MCF10A (CRL-10317; ATCC, USA) and MCF12A (CRL-10782; ATCC, USA) were cultured in MEBM (Lonza, Walkersville, MD) at 37°C in a humidified 5% CO_2_ incubator.

#### Mouse models

Female NOD-Scid gamma (NSG, Homozygous for Prkdc<scid>, Hemizygous for Il2rg<tm1Wjl>) (Strain #005557) and Balb/cJ (Strain#000651) (7–8 weeks old) mice were purchased from the Jackson Laboratory and housed under standard laboratory conditions. The Institutional Animal Care & Use Program of Oregon Health and Science University approved the protocol (#IP00003247). MDA-MB-231 (3 x 10^6^), MDA-MB-468 (5 x 10^6^), 4T1 (1 x 10^5^) and EMT6 (1 x 10^5^) cells were suspended in 100 μL of 1x PBS and injected subcutaneously in each mouse. Tumor growth was monitored regularly and once the tumors reached approximately around 50–100 mm^3^, treatment with SU056 (50 mg/kg body weight given by oral gavage, 5 days per week) was started. Tumor size and body weight were measured once a week. At the end of treatment, mice were euthanized via deep anesthesia followed by cervical dislocation. Tumors were excised and weighed. Tumor samples were processed for immunoblotting for different markers.

Different patient-derived xenograft (PDX) of TNBC were used in this study. The Institutional Animal Care & Use Program of Oregon Health and Science University approved the protocol (#IP00003247). SUTI151-PDX-TNBC was kindly provided by Stefanie S. Jeffrey, MD, Stanford University, CA, USA.^48^ CA1616-PDX-TNBC, CA1262-PDX-TNBC, and MA2821-PDX-TNBC were procured from the NCI Patient-Derived Models Repository (PDMR). Information of the PDX samples from NCI can be accessed at https://pdmr.cancer.gov/models/pdx.htm. 6–8 weeks old NSG (female) mice were anesthetized with isoflurane and tumor cells were xenografted into 5^th^ mammary fat pad. Post-xenograft, once tumors reached a size of 50–100 mm^3^, SU056 treatment (50 mg/kg body weight administered by oral gavage, 5 days per week) was started. At the end of treatment, mice were euthanized under deep anesthesia and tumors were surgically removed, measured, and weighed.

#### Rat models

Female Sprague-Dawley rats (Charles river laboratories, strain code: Crl:CD(SD) 001, 8–10 weeks old) were used by the eurofins lab services (Eurofins Discovery) to test the toxicity of SU056.

### Method details

#### Synthesis of SU056

Aza-podophyllotoxin derivative SU056 and biotinylated SU056 were synthesized and characterized following the procedure as previously described.^32^

(1) **SU056 Precursor:** (2-(benzo[d][1,3]dioxol-5-ylamino)ethan-1-ol)

**Figure:**
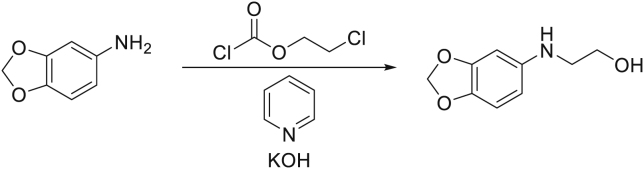

To a solution of benzo[d][1,3]dioxol-5-amine (23.0 g, 168 mmol, 1.00 equiv.) and pyridine (17.3 g, 17.6 mL, 218 mmol, 1.30 equiv.) in 300mL CH_2_Cl_2_ under nitrogen atmosphere was slowly added 2-chloroethyl carbonochloridate (25.2 g, 18.2 mL, 176 mmol, 1.05 equiv.) at 0°C. The solution turned brownish to orange and a participate was forming during the addition. After complete addition, the ice-bath was removed and the reaction mixture was stirred for 4h at room temperature, after which water was added and the resulting two phase mixture was separated. The organic phase was washed 4 times with water, dried over Na_2_SO_4_ and concentrated. The oily residue was dissolved in 260mL EtOH, KOH (37.6 g, 671 mmol, 4.00 equiv.) was added and the resulting mixture was heated to 90°C over night. On the next day the mixture was allowed to cool down to room temperature, and EtOH was evaporated under reduced pressure. The residue was dissolved in CH_2_Cl_2_, and the organic phase was extracted with 1M HCl. The aqueous phase was washed with CH_2_Cl_2_, then basified, and extracted 3x with CH_2_Cl_2_. The organic phases were combined, dried over Na_2_SO_4_, and concentrated under reduced pressure to yield the desired compound as brown solid in sufficient purity in 20% yield (6.08 g, 33.5 mmol). 1H NMR was in accordance with literature.

^1^H NMR (400 MHz, Chloroform-*d*) δ 6.69 (d, *J* = 8.3 Hz, 1H), 6.50 (d, *J* = 2.3 Hz, 1H), 6.36 (dd, *J* = 8.3, 2.3 Hz, 1H), 5.90 (s, 2H), 4.02 (s, 1H), 3.96–3.74 (m, 2H), 3.44–3.02 (m, 2H).

2. **SU056 compound:** (9-(3-fluorophenyl)-5-(2-hydroxyethyl)-6,9-dihydro-[1,3]dioxolo[4,5-g]furo[3,4-*b*]quinolin-8(5H)-one)

**Figure:**
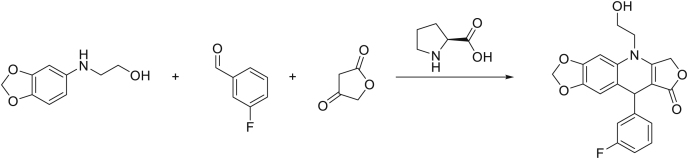

2-(benzo[d][1,3]dioxol-5-ylamino)ethan-1-ol (6.08 g, 33.5 mmol, 1.00 equiv.), 3-fluorobenzaldehyde (4.87 g, 4.16 mL, 39.2 mmol, 1.17 equiv.), furan-2,4(3H,5H)-dione (4.03 g, 40.2 mmol, 1.20 equiv.), and L-proline (386 mg, 3.35 mmol, 0.10 equiv.) were dissolved in 140 mL of dry ethanol under nitrogen atmosphere and heated to 100°C for 3h, after which TLC indicated the full consumption of the starting material. The brownish solution turned lighter to a yellow to reddish color. The solution was cooled down, which caused the product to participate from the solution. The mixture was diluted with a small amount of EtOH/CH2Cl2 (10:1), the solid was filtered of, washed and dried to obtain the desired compound as fluffy white solid in 71% yield (8.80 g, 23.8 mmol). 1H NMR was in accordance with literature.

^1^H NMR (400 MHz, DMSO-*d*_6_) δ 7.29 (td, *J* = 8.0, 6.1 Hz, 1H), 7.15–7.02 (m, 2H), 6.99–6.90 (m, 2H), 6.66 (d, *J* = 0.6 Hz, 1H), 5.99 (d, *J* = 1.0 Hz, 1H), 5.93 (d, *J* = 1.0 Hz, 1H), 5.27–4.98 (m, 3H), 4.96 (s, 1H), 3.97–3.77 (m, 1H), 3.73–3.51 (m, 3H).

#### Synthesis of biotinylated SU056

(2-(9-(3-fluorophenyl)-8-oxo-6,9-dihydro-[1,3]dioxolo[4,5-g]furo[3,4-*b*]quinolin-5(8H)-yl)ethyl 3-((2-(5-((3aS,4S,6aR)-2-oxohexahydro-1H-thieno[3,4-*d*ay]imidazol-4-yl)pentanamido)ethyl)disulfaneyl)propanoate)

**Figure:**
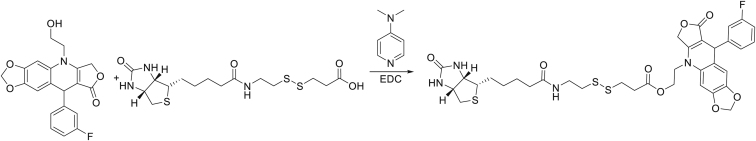

9-(3-fluorophenyl)-5-(2-hydroxyethyl)-6,9-dihydro-[1,3]dioxolo[4,5-g]furo[3,4-*b*]quinolin-8(5H)-one (45.0 mg, 122 μmol, 1.00 equiv.), 3-[2-N-(Biotinyl)aminoethyldithio]propanoic acid (49.7 mg, 122 μmol, 1.00 equiv.), EDC (28.0 mg, 146 μmol, 1.20 equiv.) and DMAP (17.9 mg, 146 μmol, 1.2 equiv.) were dissolved in dry DMF under nitrogen atmosphere. The reaction was allowed to stir for 18 h at room temperature, after which water was added and the resulting mixture was extracted with EtOAc. The combined organic phases were dried over Na_2_SO_4_, the solvent was evaporated under reduced pressure and the crude residue was purified via column chromatography (CH_2_Cl_2_/MeOH) to obtain the desired compound as a whitish solid in 19% yield (18 mg). 1H NMR was in accordance with literature.

^1^H NMR (400 MHz, Chloroform-*d*) δ 7.28–7.21 (m, 1H), 7.14–6.98 (m, 1H), 6.97–6.78 (m, 2H), 6.65–6.50 (m, 2H), 6.42–6.21 (m, 1H), 5.98 (dt, J = 10.5, 1.3 Hz, 2H), 5.38–5.23 (m, 1H), 5.15–4.88 (m, 3H), 4.71–4.30 (m, 3H), 4.14–3.91 (m, 1H), 3.90–3.79 (m, 1H), 3.75 (q, J = 7.0 Hz, 1H), 3.61–3.32 (m, 2H), 3.19 (td, J = 7.4, 4.6 Hz, 1H), 2.95 (ddd, J = 12.8, 5.0, 1.3 Hz, 1H), 2.85 (dd, J = 8.3, 6.2 Hz, 2H), 2.80–2.65 (m, 2H), 2.34–2.13 (m, 4H), 1.70 (dq, J = 14.7, 8.1, 7.4 Hz, 3H), 1.49 (q, J = 7.8 Hz, 3H), 1.36–1.13 (m, 2H).

#### Cell viability assay

MDA-MB-231 (5000 cells per well), MDA-MB-468 (6000 cells per well), SUM159 (3000 cells per well), 4T1 (2000 cells per well), EMT6 (2000 cells per well), E0771 (3000 cells per well), MCF10A (5000 cells per well) and MCF12A (5000 cells per well) cells were seeded in 96-well culture plates. On the next day, cells were treated with either DMSO or indicated concentrations of SU056 for 48 h 3-(4,5-dimethylthiazol-2-yl)-2,5-diphenyltetrazolium bromide (MTT) (0.5 mg/mL) was dissolved in 1x PBS and 50 μL were added to each well and allowed to form formazan crystals (1–2 h) followed by removal of MTT. Formazan crystals were dissolved in DMSO and plates were scanned at 570nm using a Tecan multimode plate reader.

#### Clonogenic assay

We performed a clonogenic assay to check the effect of SU056 on clonal expansion potential of MDA-MB-231, MDA-MB-468, SUM159, 4T1, EMT6, and E0771 cells on tissue culture plates. MDA-MB-231 (1000 cells), MDA-MB-468 (1000 cells), SU159 (300 cells), 4T1 (300 cells), and EMT6 (300 cells), were seeded and grown for 24 h in complete media onto 12-well culture plate. Cells were treated with the specific concentrations of SU056 for 7–10 days in complete media. Plates were processed for crystal violet staining, and the number of colonies was counted for each well. Colonies were scanned for confluency area using a Tecan multimode plate reader.

#### Cell cycle analysis

TNBC cells (MDA-MB-231, MDA-MB-468, and SUM159) were seeded (40,000 cells per well) in 12 well plates and grown in regular conditions. The next day, cells were treated with DMSO (control) and indicated SU056 concentrations. At the end of each time point, cells were processed for PI staining as described earlier.^49^ Synchronization of cells in G2/M phase was done using nocodazole (200 nM for 16 h) followed by SU056 treatment for 24 h. Cell cycle distribution was then analyzed by flow cytometry using a BD FACS Calibur (BD, USA).

#### Apoptosis assay

Apoptotic cell death induction in MDA-MB-231 and MDA-MB-468 cells in the presence of 1 and 2 μM SU056 was quantified using the Annexin V-FITC Apoptosis Assay kit from Thermo fisher scientitic, USA (Catalog number: BMS500FI-100), using the manufacturer’s protocol. Following treatment, cells were harvested by trypsinization and then washed with 1x PBS and 1x binding buffer. Subsequently, cells were resuspended in 1x binding buffer and added 5 μL of Annexin V, and then incubated for 15 min at room temperature. After incubation, cells were collected by centrifugation and resuspended in 1x binding buffer. Next, 5 μL of propidium iodide staining solution were added and incubated for 10 min at room temperature. Cells were analyzed by flow cytometry using BD FACS Calibur (BD, USA).

#### Pharmacokinetics study of SU056

Female C57BL/6J mice of 6–7 weeks were purchased from Jackson Laboratory and housed under standard laboratory conditions. SU056 (20 mg/kg) was given by oral gavage. Blood (200–300 μL) was collected retro-orbitally in EDTA-treated tubes after 15, 30, 60, 120, 240 and 360 min of SU056 administration. Blood plasma collected by centrifugation (7000 rpm; 10 min). The concentration of SU056 in plasma was analyzed using an Agilent 6490 iFunnel triple quadrupole mass spectrometer, coupled with an Agilent 1290 infinity II UHPLC. A ZORBAX C18 analytical column (Eclipse Plus, 2.1 × 50 mm, 1.8 μm particle size) was employed in the study. To create the mobile phase, a solution was prepared with 60% water buffered with 0.1% formic acid and 4mM ammonium formate, along with a solution of 40% acetonitrile buffered with 0.1% formic acid. The mobile phase flow rate was set to 0.4 mL/min and the column temperature was adjusted to 30°C. Positive ion mode was selected for operating the electrospray ionization source. Mass spectrometer parameters were optimized as: source temperature 550°C, nebulizer gas (nitrogen) 20 psi, ion spray (IS) voltage 5000 V, collision energy 21 V. Multiple reaction monitoring (MRM) method was used for the detection of SU056 and an internal standard (IS), 4-(2-hydroxyethyl)-6-methoxy-9-phenyl-4,9-dihydrofuro[3,4-*b*]quinolin-1(3H)-one, a similar analogue of SU056. The precursor ion [M + H]+ of SU056 was observed at m/z 370.0, while its product ion was detected at m/z 274.2. The precursor ion [M + H]+ was observed at m/z 338.1, while the product ion for IS was observed at m/z 260. The calibration curve, created with known concentrations of SU056 and IS, enabled the calculation of unknown concentrations of SU056 in plasma at different time points. A 5 μL plasma was taken from each sample and mixed with a 10 μL of IS solution and 990 μL of MS-grade acetonitrile then vortexed for 30 s followed by a 5 min incubation at RT. The mixture was subjected to centrifugation at 11,000 RPM for 15 min at 4°C, leading to the collection of the supernatant which was further cleaned by re-centrifugation. The concentration of SU056 was determined using the HPLC/MS MRM method with each of the three protein-free plasma fractions.^32^

#### Multi-kinase inhibition profiling of SU056

The multi-kinase inhibition profile of SU056 was determined via the KINOMEScan screening platform (Eurofins discovery). The screening was performed at 10 μM of SU056. The kinase dendrogram was developed using the TREEspot interaction maps online application (Eurofins Discovery). The results are expressed as residual activity (% Control).

#### *In vitro* pharmacology profiling of SU056

The compound SU056 was tested for its radioligand binding and enzyme and uptake characteristics by Eurofins lab services (Eurofins discovery). The screening was performed at 10 μM of SU056. The binding was calculated as % inhibition of the binding of a radioactively labeled ligand specific to each target. The SU056 enzyme inhibition effect was determined as % inhibition of control enzyme activity. The results were considered significant if SU056 showed inhibition or stimulation higher than 50%.

#### Acute oral toxicity

The acute oral toxicity of SU056 was evaluated by Eurofins lab services (Eurofins discovery). The oral toxicity study was performed in female rats and mice for multiple escalating doses (100 mg/kg, 200 mg/kg, 400 mg/kg). Rats and mice were monitored for their behavioral, neurological and autonomic signs after administration of SU056. The response was recorded as 0; no adverse effect, ±1 mild adverse effect, ±2, moderate adverse effect and ±3 severe adverse effect.

#### Global proteome profiling

MDA-MB-231, MDA-MB-468, and MCF12A cells were grown to 80% confluence and then treated with DMSO (vehicle control) or SU056 (1 μM) for 12 h. Cells were washed twice with phosphate-buffered saline (PBS), harvested by scaping, and centrifuged. Cells were lysed by vortexing in 100 mM triethylammonium bicarbonate (TEAB, Thermo Fisher Scientific) in 1% sodium dodecyl sulfate (SDS). The lysate was centrifuged, and the supernatant was retained. A bicinchoninic acid assay (BCA, Thermo Scientific, Rockville, IL) was used to measure protein concentration and 50 μg of protein per sample was subjected to disulfide bond reduction using 10 mM TCEP (Sigma Aldrich) for 1 h at 55°C. Cysteine resides were then alkylated with iodoacetamide (Sigma Aldrich) for 30 min at room temperature in the dark. Following overnight protein precipitation with acetone at −20°C, proteins were pelleted by centrifugation, redissolved in TEAB (50 mM) and digested with trypsin (Thermo Scientific) at 37°C overnight. Six-plex tandem mass tags (TMT) were brought to room temperature and dissolved in 41 μL anhydrous acetonitrile. 20.5 μL of reagent was added to each sample followed by a 1 h incubation at room temperature with subsequent quenching with 8 μL of 5% hydroxylamine (Thermo Fisher Scientific). Equal protein amounts of each sample were combined for subsequent LC-MS/MS analysis. All samples were analyzed in triplicate.

Using a Dionex Ultimate Rapid Separation Liquid Chromatography system (Thermo Fisher Scientific), a 3 μL injection of the combined TMT-labeled peptide solution was loaded onto a PepMap 100 C18 trap column (Thermo Fisher Scientific) at a rate of 5 μL/min for 10 min. Then, the TMT-labeled peptides were separated by reversed-phase chromatography on a 25 cm long C18 analytical column (New Objective) packed in house with Magic C18 AQ resin (Michrom Bioresources) by changing the mobile phase A (0.1% formic acid in water) and mobile phase B (0.1% formic acid in acetonitrile) ratio. The chromatography program consisted of holding at 2% B for the first 10 min, slowly ramping to 35% B over 100 min, followed by an increase to 85% B over 2 min with a 7 min hold time. The analytical column was re-equilibrated for 20 min with 2% B prior to the next sample injection. The flow rate was set to 0.6 μL/min throughout the gradient and each sample was analyzed in triplicate. The eluted TMT-labeled peptides were ionized using 1.8 kV on a Flex Ion source coupled to an LTQ-Orbitrap Elite mass spectrometer (Thermo Fisher Scientific). The top eight most abundant ions per MS1 scan were selected for higher energy dissociation (HCD) in a data-dependent fashion with a collisional energy set to 35 eV. The MS1 scan mass resolution was set at 30,000, FT AGC target at 1e6, and the scan mass range at 400–1800 m/z. For the MS2 scans, the mass range was set at 110–2000 m/z with a mass resolution of 30,000 and the AGC target set at 3e4 with dynamic exclusion enabled for 30 s.

Raw LC-MS/MS data was analyzed using MaxQuant^50^ searching against the Swiss-Prot human reference proteome database (20,626 entries as of 2020). Proteins were identified and quantified based on matching peptides found in each individual MS2 spectrum, using a sum-based bootstrapping algorithm on the TMT6plex reporter ion intensity after correcting for isotope impurities. The database search included the digestion of proteins by trypsin with a maximum of two missed cleavages and used a tolerance of 0.5 Da for-precursor masses and 10 ppm for fragment masses. The analysis also accounted for the fixed modification of cysteine carbamidomethylating, lysine and N-terminal TMT6plex, and variable methionine oxidation. All quantitative information was expressed in terms of Z-scores at the protein level, which were calculated by comparing the log_2_ ratios of the reporter ion intensities to the average of the control cell lines and were weighted using the WSPP model^51^ at the spectrum level before being rescaled and standardized to a normal distribution with a mean of 0 and a standard deviation of 1. The validity of the null hypothesis was carefully checked at the spectrum, peptide, and protein levels by plotting the cumulative distributions. The final statistical comparisons were performed using a Student’s t-test. The complete list of proteins and the statistical analysis can be found in Table S1.

To better understand the molecular mechanism underlying the protein changes detected (*p-value* < 0.05), we used the standalone version of FunRich^52^ to perform a gene enrichment analysis according to Reactome Pathways DB. The list of categories and their corresponding mapped proteins could be found in Table S2.

#### Cellular thermal shift assay (CETSA)

MDA-MB-231 cells treated with vehicle control (DMSO) or SU056 were analyzed by CETSA.^53^ Cells were grown to 70–80% confluency and then treated with either DMSO (vehicle control) or SU056 (1 μM) for 1.5 h. Cells were then washed twice with PBS and divided into 10 PCR tubes (1 x 10^6^ cells/tube in 100 μL PBS). The tubes incubated at different temperatures (37, 41, 44, 47, 50, 53, 56, 59, 63, 67°C) for 3 min in a thermal cycler, followed by 2 min at room temperature. The tubes were snap-frozen in liquid nitrogen, the cells were lysed using a freeze/thaw cycle, and the soluble and insoluble fractions were separated by centrifugation at 14,000 RPM for 30 min at 4°C. Equal amounts of soluble fraction for each temperature of both groups was then labeled using 10-plex TMT using the manufacturer’s protocol (Thermo Fisher Scientific). All samples were analyzed by LC-MS/MS in triplicate.

A Dionex Ultimate Rapid Separation Liquid Chromatography system (Thermo Fisher Scientific) was used to load 3 μL of the combined TMT-labeled peptides onto a PepMap C18 trap column (Thermo Fisher Scientific) with a flow rate set at 5 μL/min for 10 min. Tryptic peptides were separated by reversed-phase chromatography on a 25 cm long C18 analytical (New Objective) packed in-house with BEH C18, 130 Å, 1.7 μm particle size (Waters). A column heater (MSWIL) was used to heat the column to a temperature of 60°C. Peptides were eluted by changing the mixture of mobile phase A (0.1% formic acid in water) and mobile phase B (0.1% formic acid in acetonitrile). The gradient program consisted of holding mobile phase B at 2% for the first 6 min, slowly ramping up to 35% over the next 104 min, followed by an increase to 85% over 5 min with a 5 min hold. The analytical column was re-equilibrated 10 min prior to the next sample injection. The flow rate throughout the gradient was set to 0.3 μL/min and each sample was analyzed in triplicates. Eluted peptides were analyzed using an Orbitrap Eclipse Tribrid mass spectrometer (Thermo Fisher Scientific). The cycle time was set at Top-speed for 3 s with an MS1 mass scan range of 375–1600 m/z and a mass resolution of 120,000. The normalized AGC target was set to custom with a target value of 250. The precursor fit filter was enabled with 50% threshold and 0.7 m/z fit window. Dynamic exclusion was enabled for 60 s with a mass tolerance of 10 ppm. The most abundant precursor ions were subject to collision induced dissociation (CID) with a collisional energy set to 35 eV and detected in the ion trap using Turbo scan rate. The mass range scan was set to normal and the AGC target value set to standard. The maximum injection time for MS2 was set to 35 ms. The Real-Time-Search algorithm was enabled with search time of 100 ms for peptide identification against the Swiss-Prot database reference human proteome (2020, 20,366 entries). A fixed modification of Carbamidomethyl and TMT6plex was added on cysteine and lysine residues and a variable modification on methionine for peptide identification. Identified TMT-labeled peptides were selected for MS3 by enabling synchronous precursor selection (SPS) algorithm using a scan mass range of 400–1600. The maximum number of SPS precursor ions allowed was set to 10 with an isolation window of 1.2 m/z. Selected precursor ions were subject to higher energy dissociation (HCD) with a collision energy of 65% in the Orbitrap detector. The mass resolution was set to 60,000 and a scan mass range at 100–500 m/z. The normalized AGC target was set to custom with a target value of 200.

In the second CETSA experiment with MDA-MB-231- YB-1 knockdown cells, the Orbitrap Eclipse Tribrid mass spectrometer parameters consisted of setting the cycle time at Top-speed at 1 s, the mass scan range set between 375 and 1800 m/z, and a mass resolution of 50,000. The normalized AGC target and maximum injection time was set to standard and auto respectively. The precursor ions were selected from highest to lowest charge state, sorted by the lowest m/z, and fragmented using Higher Energy Collisional Dissociation (HCD) with a fixed collisional energy of 38%. Dynamic exclusion was enabled for 60 s with a mass tolerance of ±10 ppm. The normalized AGC target set to 200% with a maximum injection time of 86 ms. MS2 fragments were detected in the Orbitrap with a mass resolution of 50,000 and scan range window of 110–1800 m/z.

Proteins were quantified using individual spectra matched peptides based on a sum-based bootstrap algorithm, which used the TMT reporter ion intensity after correcting for isotope impurities with Proteome Discoverer 2.4. The protein levels were compared between the vehicle and SU056 treated samples. The lowest temperature was used as a reference to calculate the log_2_ ratio of the soluble fraction signal at each temperature, and the signal at each temperature was compared to the highest temperature to estimate the percentage of signal lost in the soluble fraction. Finally, the data were fitted to the S-curves minimizing the sum of squares difference between the original and Boltzmann-adjusted curves using a brute-force algorithm in R. The melting point differences between the fitted curves (treatment – control) with correlations above 0.7, *p*-values less than 0.01, and their calculated thermal shift are consistent in all measures performed were considered specific interactors of the drug.

#### Immunoblot assay

MDA-MB-231, MDA-MB-468, and SUM159 cells were grown to 60–70% confluency and treated with DMSO and SU056 (1 μM) for 12 and 24 h. After the specific treatment time was over, total cell lysates were prepared in non-denaturing lysis buffer. For protein lysate from tumors: randomly selected frozen sample; three from vehicle and SU056 treated group were homogenized in non-denaturing lysis buffer at 4°C for 5 min. 10 μg of protein lysate per sample was resolved on 8–16% SDS-PAGE. Membranes were probed with specific primary antibodies followed by detection with HRP-conjugated appropriate secondary antibodies, visualized by enhanced chemiluminescence detection system, and imaged using an iBright imaging system (Thermo Fisher, USA).

#### Immunoprecipitation

MDA-MB-231 and MDA-MB-468 cells with and without SU056 (1 μM) treatment for 6 and 12 h respectively and were lysed using M-PER lysis solution (Thermo Scientific, #78503), supplemented with Halt protease and phosphatase inhibitor cocktail (Thermo Scientific, #78440). Precleaned 300 μg protein lysate was incubated overnight at 4°C with 3 μg YB-1 (Abcam, #ab76149) on nutating rocker. The antibody was pulled down using 20 μL Protein A/G magnetic beads (Thermo scientific #88802). After three washings, beads were resuspended in 2X SDS sample buffer followed by heating at 90°C–100°C for 5 min. Samples were resolved and probed with indicated antibodies as described in immunoblotting assay.

#### Pulldown assay with biotinylated SU056

Protein pulldown assay was performed using biotinylated SU056. MDA-MB-231 and MDA-MB-468 cells were treated with 2 μM biotinylated SU056 for 2 h. After 2 h of treatment, cells were collected and lysed using M-PER lysis solution (Thermo Scientific, #78503), supplemented with Halt protease and phosphatase inhibitor cocktail (Thermo Scientific, #78440). Protein lysate from Biotinylated SU056 treated cells were treated with RNase (10 μg/mL) for 15 min at 37°C. 600 μg protein was incubated with magnetic conjugate streptavidin bead (CST, #5947) at room temperature on rocker for 30 min. Biotin-streptavidin conjugates were pulled down and washed using a magnetic rack. After three washes, beads were resuspended in 2X SDS sample buffer followed by heating at 90°C–100°C for 5 min. Samples were resolved and probed with anti-YB-1 and anti-RPL11 antibodies as described in immunoblotting. Samples of protein lysates from MDA-MB-231 and MDA-MB-468 without biotinylated SU056 (input), samples from pulldown using only biotin and only streptavidin beads were used as experimental controls.

#### Polysome profiling

1 x 10^6^ MDA-MB-231 cells were plated in 100 mm culture plate and allowed to be attached for 24 h. Next day, cells were treated with vehicle or SU056 (1 μM) for 12 h in regular culture medium. Next, cells were incubated with cycloheximide (10 μg/mL) for 5 min at 37°C. Cells were then washed twice in 1x PBS with 10 μg/mL cycloheximide and collected by trypsinization and snap freeze in liquid nitrogen. Cell pellet was lysated in 250 μL polysome lysis buffer (5 mM Tris-HCl [pH 7.4], 1.5 mM KCl, 2.5 mM MgCl_2_, 0.1 mg/mL cycloheximide, 100 U/mL RNasin, 1× EDTA-free Complete protease inhibitor cocktail, 2 mM DTT, 0.5% Triton X-100, 0.5% deoxycholate). Lysate were quantified taking absorbance at 260 nm using Nanodrop and loaded on a linear 15%–50% sucrose gradient. The gradients were ultracentrifuged SW41Ti rotor (Beckman) for 1 h and 30 min at 180,000 g at 4°C in Beckman Optima LE-80K Ultracentrifuge. The gradients were fractionated in 1 mL volume fractions with continuous monitoring absorbance at 254 nm using an ISCO UA-6 UV detector at Immagina Biotechnology, Pergine, Italy.

### Quantification and statistical analysis

Statistical analyses of each experiment were calculated using GraphPad Prism 9.0 software. Each data in figures is represented as a mean ± SD, where ∗ indicates *p* < 0.05, ∗∗ indicates *p* < 0.01, ∗∗∗ indicates *p* < 0.001 and ∗∗∗∗ indicates *p* < 0.0001. Statistical significance of the differences between control and the treated group was determined by the Student’s t test and one-way ANOVA followed by Sidak and Dunnet’s adjustment.

## Supplemental information

Document S1. Data S1–S5 and Figures S1–S5

Data S6. Raw western blots

Table S1. List of proteins identified in proteomic analysis in TNBC cells by SU056 treatment, related to Figure 4

Table S2. List of pathways identified in proteomic analysis in TNBC cells by SU056 treatment, related to Figure 4

Table S3. List of proteins whose thermal shift change is observed in MDA-MB-231 cells by SU056 treatment using CETSA assay, related to Figure 5

Table S4. List of proteins whose thermal shift change is observed in YBX1-knockdown condition by CETSA analysis, related to Figure 5

Document S2. Article plus supplemental information
