# Supplementary material for: Inhibition of protein translational machinery in triple-negative breast cancer as a promising therapeutic strategy
Source: Cell Rep Med. 2024 May 9;5(5):101552. doi: 10.1016/j.xcrm.2024.101552 (PMC11148772; doi:10.1016/j.xcrm.2024.101552)
Supplement: Document S1. Data S1–S5 and Figures S1–S5 [file mmc1.pdf]

Cell Reports Medicine, Volume 5

Supplemental information

Inhibition of protein translational machinery in triple-negative breast cancer as a promising therapeutic strategy

Arpit Dheeraj, Fernando Jose Garcia Marques, Dhanir Tailor, Abel Bermudez, Angel Resendez, Mallesh Pandrala, Benedikt Grau, Praveen Kumar, Carrsyn B. Haley, Alexander Honkala, Praveen Kujur, Stefanie S. Jeffrey, Sharon Pitteri, and Sanjay V. Malhotra

Inhibition of protein translational machinery in triple-negative breast cancer as a promising therapeutic strategy

Arpit Dheeraj[1,2,3], Fernando Jose Garcia Marques[4], Dhanir Tailor[1,2,3], Abel Bermudez[4], Angel Resendez[3], Mallesh Pandrala[1,2], Benedikt Grau[1,2], Praveen Kumar[1,2], Carrsyn B. Haley[2], Alexander Honkala[1,2], Praveen Kujur[5], Stefanie S. Jefferey[5], Sharon Pitteri[4], Sanjay V. Malhotra[1,2,3,6]

[1]Department of Cell, Development and Cancer Biology, Knight Cancer Institute, Oregon Health & Science University, Portland, OR, USA

[2]Center for Experimental Therapeutics, Knight Cancer Institute, Oregon Health & Science University, Portland, OR, USA

[3]Department of Radiation Oncology, Stanford University School of Medicine, Palo Alto, CA, USA

[4]Department of Radiology, Canary Center at Stanford for Cancer Early Detection, Stanford University School of Medicine, Palo Alto, CA, USA

[5]Department of Surgery, Stanford University School of Medicine, Palo Alto, CA, USA

[6]Lead contact

Data S1: SU056 induces cell cycle arrest and apoptotic cell death, related to Figure 1.

Data S2: Effect of SU056 treatment on body weight, metastatic potential and pharmacokinetic profiling of SU056, related to Figure 2 and 3.

Data S3: Maximum Tolerated Dose and acute oral toxicity of SU056 in female mice and SD rat, related to Figure 3.

Data S4: Kinase profiling and pharmacological parameters of SU056, related to Figure 3.

Data S5: SU056 targets translation processes in SUM159 cells and does not induce the integrated stress response pathway, related to Figure 6.

Data S1. SU056 induces cell cycle arrest and apoptotic cell death, related to Figure 1 **A)** The growth inhibitory effect of SU056 was evaluated using the MTT assay (0.5 mg/ml in 1x PBS). Normal breast epithelial cells (MCF10A and MCF12A) were plated in a 96 well plate. On the next day, cells were treated with vehicle (DMSO) alone or 0.005-50 μM of SU056 in fresh medium. IC_{50} values were determined by MTT after 48 hr. Error bars represent mean ±SD. **B)** MDA-MB-231 and MDA-MB-468 cells were treated for 12 and 24 hr and total cell lysates were prepared. 10-20 μg of protein was run on SDS-PAGE and western blot analyses were performed for stress response related and cell cycle regulatory molecules. β-actin was probed to ensure equal protein loading. **C) and D)** MDA-MB-231 and MDA-MB-468 cell cycle analysis by flow cytometry using propidium-iodide staining of G2/M phase synchronized cells induced via nocodazole treatment. SU056 treatment led to cell cycle arrest at the G2/M phase and induced cell death in TNBC cells. **E)** MDA-MB-231 **and F)** MDA-MB-468 cells were analyzed for cell death using Annexin V-FITC Apoptosis Assay kit. Cell lysates were analyzed for Bax and BCL-2 proteins. β-actin was probed to ensure equal protein loading.

Data are shown as mean ± SD. Error bars represent ± SD.
*$p < 0.05$, **$p < 0.01$, ***$p < 0.001$ and ****$p < 0.0001$. Significant difference compared with respective control by Student's t test or one-way ANOVA followed by Dunnett's test.

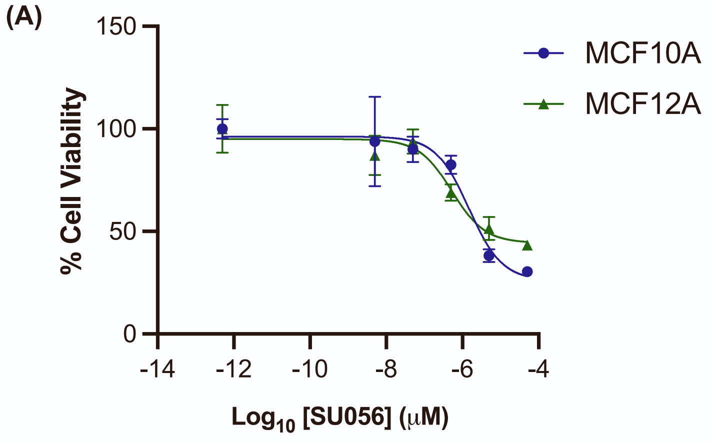

Cell line	IC_{50} (µM)
MCF10A	4.77± 1.61
MCF12A	4.65±2.25

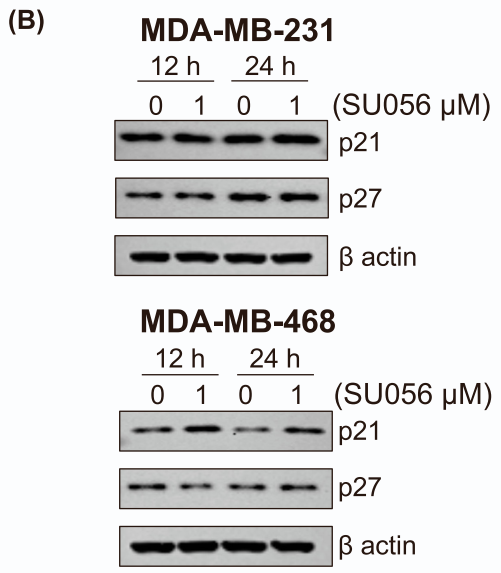

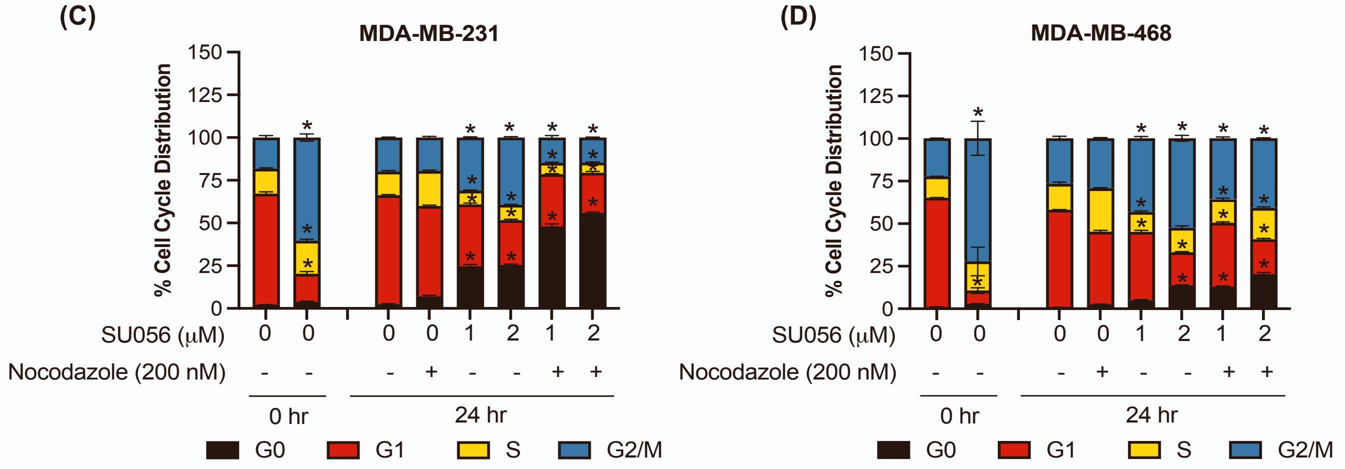

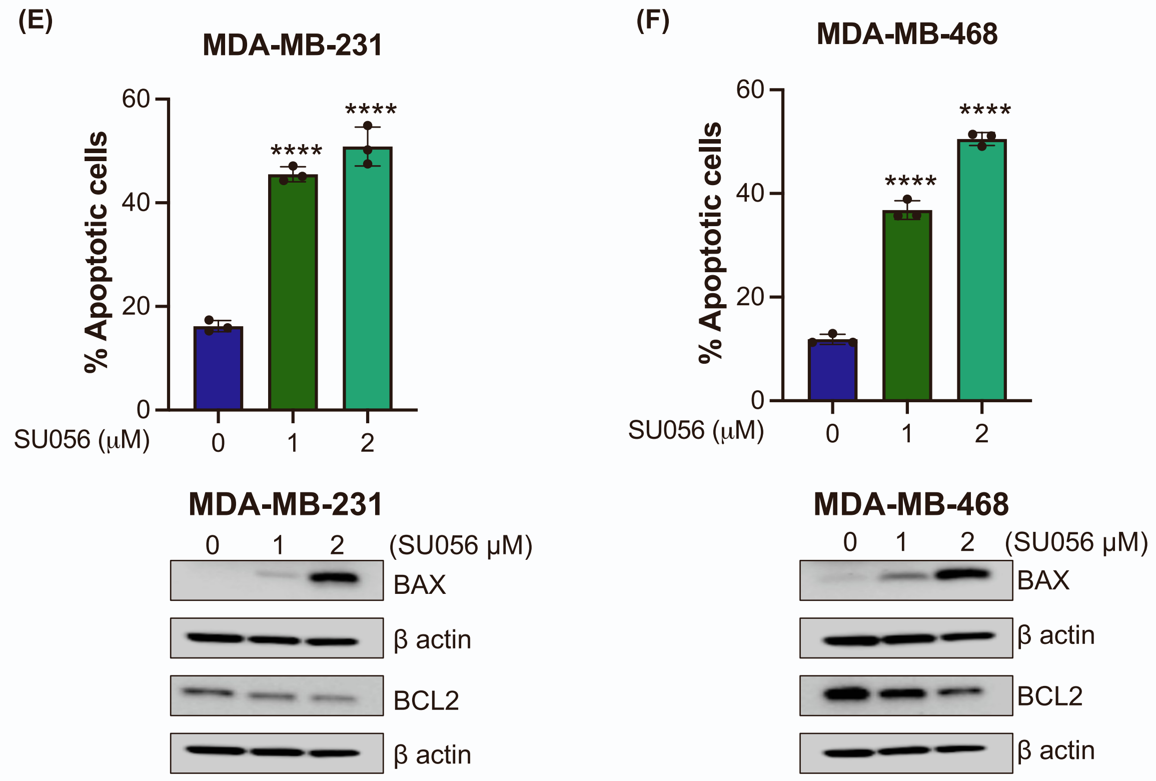

Data S2. Effect of SU056 treatment on body weight, metastatic potential and pharmacokinetic profiling of SU056, related to Figure 2 and 3

Human, mouse and PDX models of TNBC were used for tumor xenograft studies. SU056 treatment was started when tumors reached around ~25-50 mm^3 and continued until the experiment reached its 3-week endpoint. Body weight was monitored throughout the study to understand any change in dietary pattern. Body weights of **A)** MDA-MB-231, **B)** MDA-MB-468, **C)** 4T1, **D)** EMT6, **E)** CA1616-PDX-TNBC, **F)** CA1262-PDX-TNBC, **G)** MA2821-PDX-TNBC and **H)** SUTI151-PDX-TNBC over time. **I)** Tumor volume, **J)** Tumor weight and **K)** The bar graph represents lung metastatic nodules in 4T1 orthotopic models. **L)** Representative images of lungs at the end of the treatment cycle. **M)** Mice were treated with 20 mg/kg SU056 orally. C_{max} (maximum serum concentration), T_{max} (time to reach C_{max}), and $T_{1/2}$ (elimination half-life of compound). **N)** Human liver microsomes were used for the purpose of metabolic profiling. SU056 had a mean half-life of 40 minutes.

Data are shown as mean ± SD. Error bars represent ± SD.

*p < 0.05, **p < 0.01, ***p < 0.001 and ****p < 0.0001. Significant difference compared with respective control by Student’s t test or one-way ANOVA followed by Dunnett’s test.

Data S2

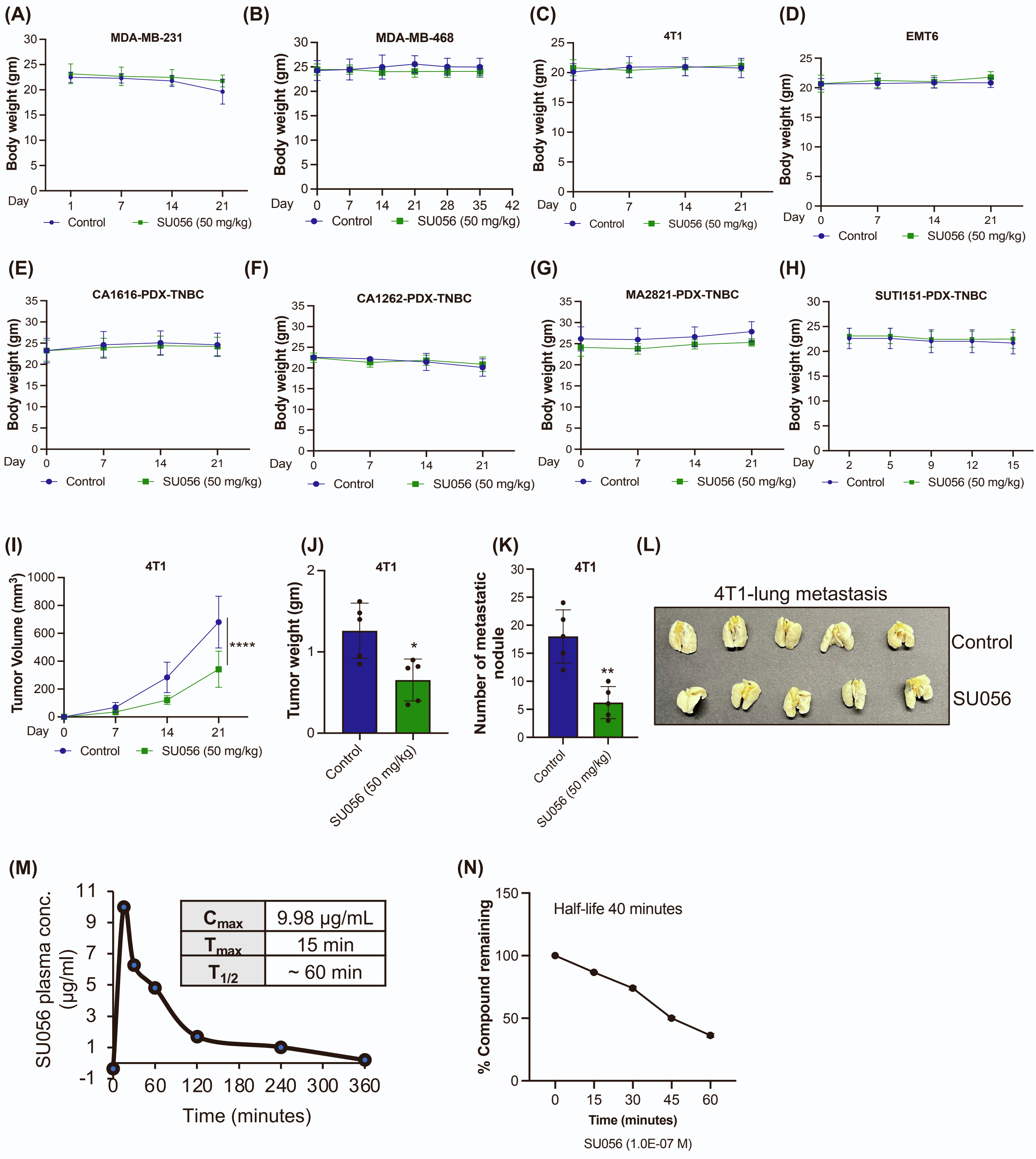
(A)
MDA-MB-231
Body weight (gm)
Day
Control
SU056 (50 mg/kg)
(B)
MDA-MB-468
(C)
4T1
(D)
EMT6
(E)
CA1616-PDX-TNBC
(F)
CA1262-PDX-TNBC
(G)
MA2821-PDX-TNBC
(H)
SUTI151-PDX-TNBC
(I)
4T1
Tumor Volume (mm^3)
****
(J)
4T1
Tumor weight (gm)
*
(K)
4T1
Number of metastatic nodule
**
(L)
4T1-lung metastasis
Control
SU056
(M)
SU056 plasma conc. (µg/ml)
Time (minutes)
C_{max} 9.98 µg/mL
T_{max} 15 min
$T_{1/2}$ ~ 60 min
(N)
Half-life 40 minutes
% Compound remaining
Time (minutes)
SU056 (1.0E-07 M)

Data S3. Maximum Tolerated Dose and acute oral toxicity of SU056 in female mice and SD rat, related to Figure 3

A) Oral administration of SU056 is well tolerated at high doses. Rat and mice were monitored for their behavior and adverse event such as death for 72 hr. **B)** Behavioral, neurologic, and anatomical signs of distress and toxicity are not affected by SU056 oral administration at 100 mg/kg, 200 mg/kg, or 400 mg/kg doses in female mice and **C)** SD rat. **D)** SU056 treatment at increasing concentrations dose not challenge the dietary behavior as reflected by change in body weight. SU056 was given at maximum concentration of 100 mg/kg, 200 mg/kg and 400 mg/kg body weight to mouse and rat and change in body weight were measured.

Data S3

(A)

Maximum Tolerated Dose, Mice						
Toxicity						
Strain: ICR (Female)						
Compound	Dose (mg/kg)	Toxicity (death/test)				
		15 min	1 hr	24 hr	48 hr	72 hr
Vehicle (5% DMSO/45% PEG300/ 50% Saline)	-	0/3	0/3	0/3	0/3	0/3
SU056	100	0/3	0/3	0/3	0/3	0/3
	200	0/3	0/3	0/3	0/3	0/3
	400	0/3	0/3	0/3	0/3	0/3
Strain: Female SD rats						
Compound	Dosage (mg/kg)	Toxicity (death/test)				
		15 min	1 hr	24 hrs	48 hrs	72 hrs
Vehicle (5% DMSO/45% PEG300/ 50% Saline)	-	0/3	0/3	0/3	0/3	0/3
SU056	100	0/3	0/3	0/3	0/3	0/3
	200	0/3	0/3	0/3	0/3	0/3
	400	0/3	0/3	0/3	0/3	0/3

(B)

Maximum Tolerated Dose, Mice												
Behavioral, Neurologic and Autonomic signs												
Observation time: 60 min Strain: ICR (Female)												
Treatment	Vehicle (5% DMSO/45% PEG300/ 50% Saline)			SU056								
Dosage	-			100			200			400		
	1	2	3	1	2	3	1	2	3	1	2	3
**Behavioral profile**												
Irritability	-	-	-	-	-	-	-	-	-	-	-	-
Hyperactivity	-	-	-	-	-	-	-	-	-	-	-	-
Inc.Startle	-	-	-	-	-	-	-	-	-	-	-	-
Inc.Touch	-	-	-	-	-	-	-	-	-	-	-	-
Dec.Startle Response	-	-	-	-	-	-	-	-	-	-	-	-
Dec.Touch Response	-	-	-	-	-	-	-	-	-	-	-	-
Inc.Exploration	-	-	-	-	-	-	-	-	-	-	-	-
Dec.Exploration	-	-	-	-	-	-	-	-	-	-	-	-
Pinna	-	-	-	-	-	-	-	-	-	-	-	-
Placing	-	-	-	-	-	-	-	-	-	-	-	-
**Neurologic profile**												
Tremor	-	-	-	-	-	-	-	-	-	-	-	-
Dec. Spont. Activity	-	-	-	-	-	-	-	-	-	-	-	-
Straub Tail	-	-	-	-	-	-	-	-	-	-	-	-
Reactivity	-	-	-	-	-	-	-	-	-	-	-	-
Righting	-	-	-	-	-	-	-	-	-	-	-	-
Ataxia	-	-	-	-	-	-	-	-	-	-	-	-
Convulsion C.T.C-T	-	-	-	-	-	-	-	-	-	-	-	-
Low Limb Post	-	-	-	-	-	-	-	-	-	-	-	-
Abdominal Tone	-	±	-	-	-	-	-	-	±	-	-	±
Limb Tone	-	-	-	-	-	-	-	-	-	-	-	-
Grip Strength	-	-	-	-	-	-	-	-	-	-	-	-
**Autonomic profile**												
Skin Color	-	-	-	-	-	-	-	-	-	-	-	-
Respiration	-	-	-	-	-	-	-	-	-	-	-	-
Salivation F.V.	-	-	-	-	-	-	-	-	-	-	-	-
Lacrimation	-	-	-	-	-	-	-	-	-	-	-	-
Diarrhea	-	-	-	-	-	-	-	-	-	-	-	-
Body Temperature	-	-	-	-	-	-	-	-	-	-	-	-
Piloerection	-	-	-	-	-	-	-	-	-	-	±	-
Inc.Palpebral Size	-	-	-	-	-	-	-	-	-	-	-	-
Dec. Palpebral Size	-	-	-	-	-	-	-	-	-	-	-	-
Others	-	-	-	-	-	-	-	-	-	-	-	-
Death	-	-	-	-	-	-	-	-	-	-	-	-

(C)

Maximum Tolerated Dose, Rat												
Behavioral, Neurologic and Autonomic signs												
Observation time: 1 hr Strain: Female SD rats												
Treatment	Vehicle (5% DMSO/45% PEG300/ 50%			SU056								
Dosage	-			100 mg/kg			200 mg/kg			400 mg/kg		
Animal No.	1	2	3	1	2	3	1	2	3	1	2	3
**Behavioral Profile**	Response 0, ±1, ±2, ±3											
Alertness	0	0	0	0	0	0	0	0	0	0	0	0
Passivity	0	0	0	0	0	0	0	0	0	0	0	0
Stereotypy	0	0	0	0	0	0	0	0	0	0	0	0
Vocalization	0	0	0	0	0	0	0	0	0	0	0	0
Transfer Reactivity	0	0	0	0	0	0	0	0	0	0	0	0
Touch Escape	0	0	0	0	0	0	0	0	0	0	0	0
Tail - Pinch	0	0	0	0	0	0	0	0	0	0	0	0
Toe - Pinch	0	0	0	0	0	0	0	0	0	0	0	0
Pinna Reflex	0	0	0	0	0	0	0	0	0	0	0	0
Startle Response	0	0	0	0	0	0	0	0	0	0	0	0
Visual Placing	0	0	0	0	0	0	0	0	0	0	0	0
**Neurologic Profile**												
Body Elevation	0	0	0	0	0	0	0	0	0	0	0	0
Limb Position	0	0	0	0	0	0	0	0	0	0	0	0
Tail Elevation	0	0	0	0	0	0	0	0	0	0	0	0
Limb Tone	0	0	0	0	0	0	0	0	0	0	0	0
Grip Strength	0	0	0	0	0	0	0	0	0	0	0	0
Body Tone	0	0	0	0	0	0	0	0	0	0	0	0
Abdominal Tone	0	0	0	0	0	0	0	0	0	0	0	0
Change in Gait	0	0	0	0	0	0	0	0	0	0	0	0
Catalepsy	0	0	0	0	0	0	0	0	0	0	0	0
Righting Reflex	0	0	0	0	0	0	0	0	0	0	0	0
Twitches	0	0	0	0	0	0	0	0	0	0	0	0
Convulsion	0	0	0	0	0	0	0	0	0	0	0	0
**Autonomic Profile**												
Excretion (Urination, Diarrhea)	0	0	0	0	0	0	0	0	0	0	0	0
Secretion (Salivation, Lacrimation)	0	0	0	0	0	0	0	0	0	0	0	0
Skin Color (Blanch, Flush, Cyanosis)	0	0	0	0	0	0	0	0	0	0	0	0
Respiration (Fast, Slow, Deep, Irregular)	0	0	0	0	0	0	0	0	0	0	0	0
Palpebral Size	0	0	0	0	0	0	0	0	0	0	0	0
Piloerection	0	0	0	0	0	0	0	0	0	0	0	0
Body Temperature	0	0	0	0	0	0	0	0	0	0	0	0
Death	-	-	-	-	-	-	-	-	-	-	-	-

(D)

Maximum Tolerated Dose						
Body Weight						
Strain: ICR (Female)						
Compound	Dose (mg/kg)	No.	Body Weight (gm)			
			0 hr	24 hr	48 hr	72 hr
Vehicle (5% DMSO/45% PEG300/ 50% Saline)	-	1	23	23	24	24
		2	23	23	23	23
		3	23	24	23	26
		Mean	23.0	23.3	23.3	24.3
		SEM	0.0	0.3	0.3	0.9
SU056	100	1	23	22	23	24
		2	23	23	23	24
		3	23	24	24	25
		Mean	23.0	23.0	23.3	24.3
		SEM	0.0	0.6	0.3	0.3
	200	1	22	23	24	23
		2	24	24	23	24
		3	25	25	25	26
		Mean	23.7	24.0	24.0	24.3
		SEM	0.9	0.6	0.6	0.9
	400	1	22	22	23	24
		2	22	23	23	24
		3	24	23	24	25
		Mean	22.7	22.7	23.3	24.3
		SEM	0.7	0.3	0.3	0.3
Strain: Female SD rats						
Vehicle (5% DMSO/45% PEG300/ 50% Saline)	-	1	212	221	225	228
		2	210	213	218	225
		3	205	211	214	226
		Mean	209.0	215.0	219.0	226.3
		SEM	2.1	3.1	3.2	0.9
SU056	100	1	203	208	211	225
		2	220	224	226	232
		3	215	218	220	232
		Mean	212.7	216.7	219.0	229.7
		SEM	5.0	4.7	4.4	2.3
	200	1	206	215	213	216
		2	207	206	212	219
		3	200	203	208	210
		Mean	204.3	208.0	211.0	215.0
		SEM	2.2	3.6	1.5	2.6
	400	1	200	196	203	211
		2	203	199	212	216
		3	209	205	214	223
		Mean	204.0	200.0	209.7	216.7
		SEM	2.6	2.6	3.4	3.5

Data S4. Kinase profiling and pharmacological parameters of SU056, related to Figure 3 **A)** Dendrogram representation of the kinase inhibition profiles of SU056. **B)** The bar graph represents the top 12 kinases inhibited by SU056 in KINOMEScan profiling. **C)** Screening for IC_{50} value for 5 different kinase (PIK3C3, DYRK1B, FLT3, FLT3-D835Y, and mTOR). **D)** Enzyme and uptake assay with SU056. The bar graph shows inhibition or stimulation in the binding assay by SU056 with respect to control specific binding. **E)** *In vitro* radioligand binding assay performed with SU056 (10 μM).

Data S4

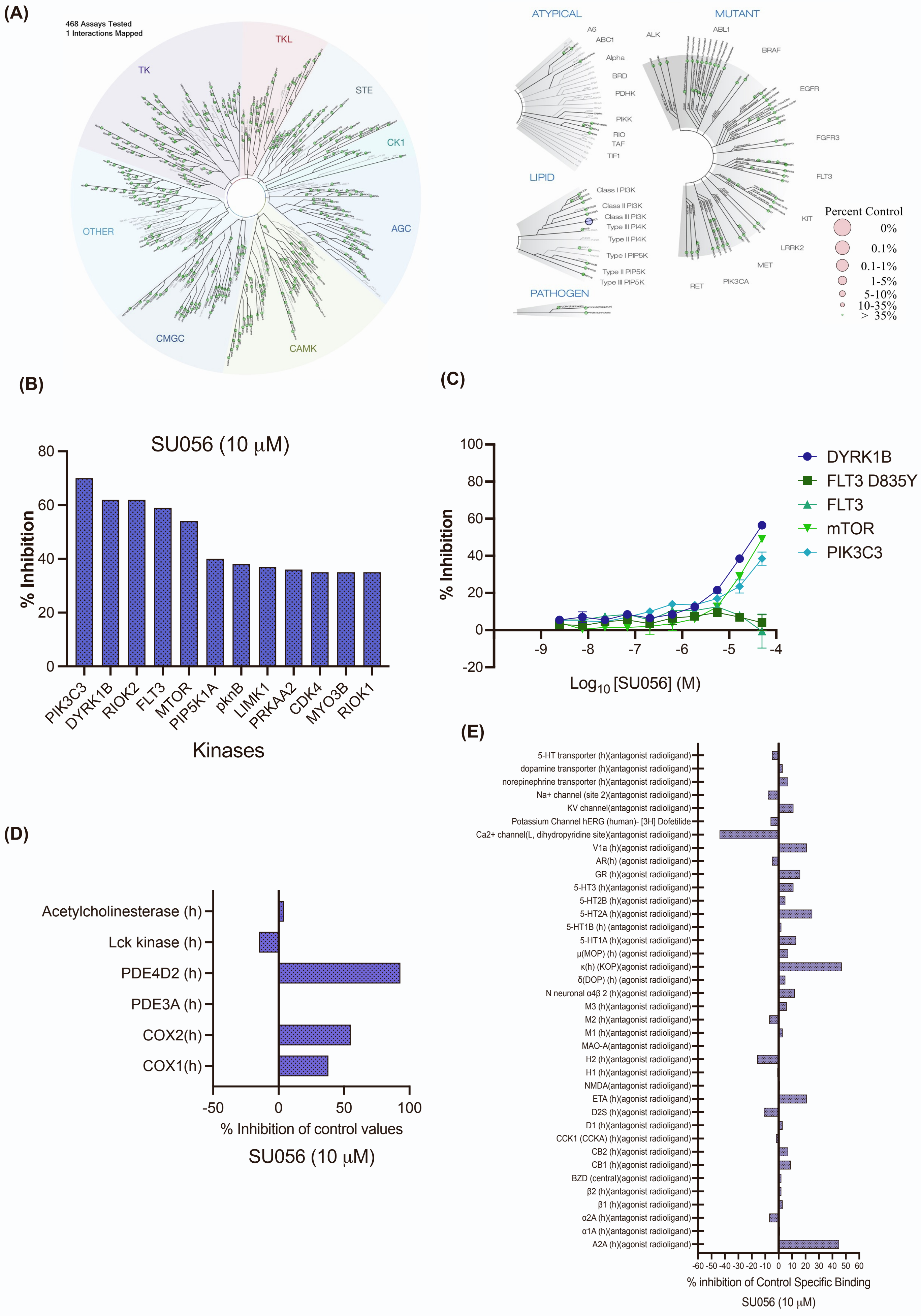
(A)
468 Assays Tested
1 Interactions Mapped
TKL
TK
STE
CK1
AGC
OTHER
CMGC
CAMK
ATYPICAL
MUTANT
A6
ABC1
Alpha
BRD
PDHK
PIKK
RIO
TAF
TIF1
ALK
ABL1
BRAF
EGFR
FGFR3
FLT3
KIT
LRRK2
MET
PIK3CA
RET
LIPID
Class I PI3K
Class II PI3K
Class III PI3K
Type III PI4K
Type II PI4K
Type I PIP5K
Type II PIP5K
Type III PIP5K
PATHOGEN
Percent Control
0%
0.1%
0.1-1%
1-5%
5-10%
10-35%
> 35%
(B)
SU056 (10 µM)
% Inhibition
0
20
40
60
80
PIK3C3
DYRK1B
RIOK2
FLT3
MTOR
PIP5K1A
pknB
LIMK1
PRKAA2
CDK4
MYO3B
RIOK1
Kinases
(C)
% Inhibition
100
80
60
40
20
0
-20
-9
-8
-7
-6
-5
-4
Log10 [SU056] (M)
DYRK1B
FLT3 D835Y
FLT3
mTOR
PIK3C3
(D)
Acetylcholinesterase (h)
Lck kinase (h)
PDE4D2 (h)
PDE3A (h)
COX2(h)
COX1(h)
-50
0
50
100
% Inhibition of control values
SU056 (10 µM)
(E)
5-HT transporter (h)(antagonist radioligand)
dopamine transporter (h)(antagonist radioligand)
norepinephrine transporter (h)(antagonist radioligand)
Na+ channel (site 2)(antagonist radioligand)
KV channel(antagonist radioligand)
Potassium Channel hERG (human)- [3H] Dofetilide
Ca2+ channel(L, dihydropyridine site)(antagonist radioligand)
V1a (h)(agonist radioligand)
AR(h) (agonist radioligand)
GR (h)(agonist radioligand)
5-HT3 (h)(antagonist radioligand)
5-HT2B (h)(agonist radioligand)
5-HT2A (h)(agonist radioligand)
5-HT1B (h) (antagonist radioligand)
5-HT1A (h)(agonist radioligand)
µ(MOP) (h) (agonist radioligand)
κ(h) (KOP)(agonist radioligand)
δ(DOP) (h) (agonist radioligand)
N neuronal α4β 2 (h)(agonist radioligand)
M3 (h)(antagonist radioligand)
M2 (h)(antagonist radioligand)
M1 (h)(antagonist radioligand)
MAO-A(antagonist radioligand)
H2 (h)(antagonist radioligand)
H1 (h)(antagonist radioligand)
NMDA(antagonist radioligand)
ETA (h)(agonist radioligand)
D2S (h)(agonist radioligand)
D1 (h)(antagonist radioligand)
CCK1 (CCKA) (h)(agonist radioligand)
CB2 (h)(agonist radioligand)
CB1 (h)(agonist radioligand)
BZD (central)(agonist radioligand)
β2 (h)(antagonist radioligand)
β1 (h)(agonist radioligand)
α2A (h)(antagonist radioligand)
α1A (h)(antagonist radioligand)
A2A (h)(agonist radioligand)
-60 -50 -40 -30 -20 -10 0 10 20 30 40 50 60
% inhibition of Control Specific Binding
SU056 (10 µM)

Data S5. SU056 targets translation processes in SUM159 cells and does not induce the integrated stress response pathway, related to Figure 6

MDA-MB-231, MDA-MB-468, and SUM159 cells were treated for 12 and 24 hr and total cell lysates were prepared. 10-20 μg of protein was run on SDS-PAGE and western blot analyses were performed for translation-associated molecules. β-actin was probed to ensure equal protein loading. SU056 treatment inhibited protein translation-associated molecules in all TNBC models. **A)** Protein translation machinery molecules; **B)** Translation initiation factors molecules; **C)** Ribosomal large subunit; and **D)** Ribosomal small subunit proteins **E)** Stress response-associated proteins in MDA-MB-231 and MDA-MB-468 cells.

Data S5

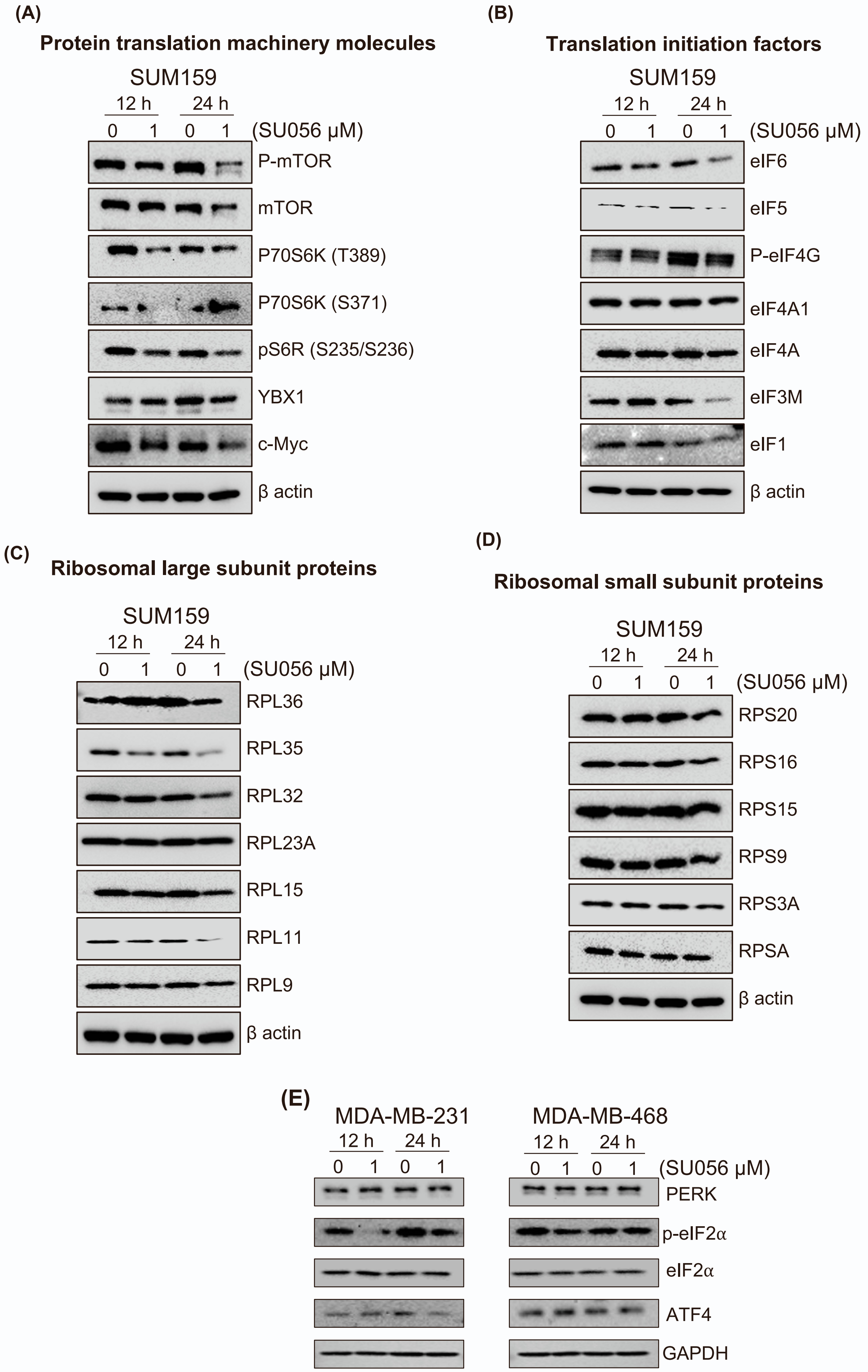
(A)
Protein translation machinery molecules
SUM159
12 h
24 h
0 1 0 1 (SU056 μM)
P-mTOR
mTOR
P70S6K (T389)
P70S6K (S371)
pS6R (S235/S236)
YBX1
c-Myc
β actin
(B)
Translation initiation factors
SUM159
12 h
24 h
0 1 0 1 (SU056 μM)
eIF6
eIF5
P-eIF4G
eIF4A1
eIF4A
eIF3M
eIF1
β actin
(C)
Ribosomal large subunit proteins
SUM159
12 h
24 h
0 1 0 1 (SU056 μM)
RPL36
RPL35
RPL32
RPL23A
RPL15
RPL11
RPL9
β actin
(D)
Ribosomal small subunit proteins
SUM159
12 h
24 h
0 1 0 1 (SU056 μM)
RPS20
RPS16
RPS15
RPS9
RPS3A
RPSA
β actin
(E)
MDA-MB-231
MDA-MB-468
12 h
24 h
0 1 0 1
0 1 0 1 (SU056 μM)
PERK
p-eIF2α
eIF2α
ATF4
GAPDH
