# Supplementary material for: Inhibition of protein translational machinery in triple-negative breast cancer as a promising therapeutic strategy
Source: Cell Rep Med. 2024 May 9;5(5):101552. doi: 10.1016/j.xcrm.2024.101552 (PMC11148772; doi:10.1016/j.xcrm.2024.101552)
Supplement: Data S6. Raw western blots [file mmc2.pdf]

Figure 1J, 1K and 1L

MDA-MB-231 and MDA-MB-468

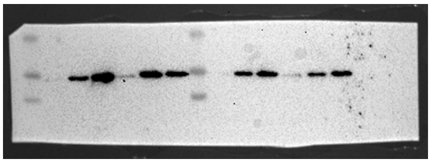

p-Histone H3

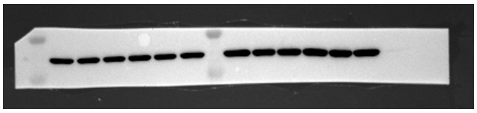

β actin

SUM159

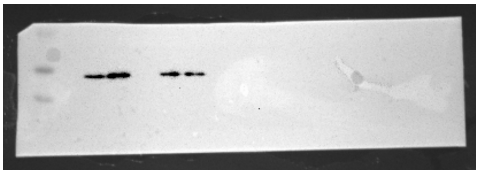

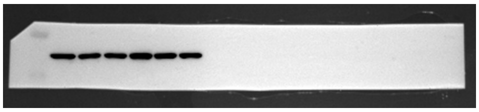

Figure 6A and Supplementary figure 9A

MDA-MB-231, MDA-MB-468 and SUM159

MDA-MB-231, MDA-MB-468 and SUM159

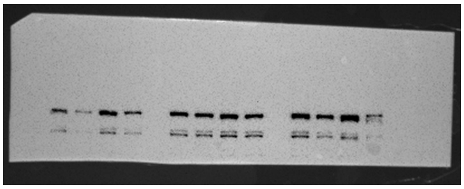
p-mTOR

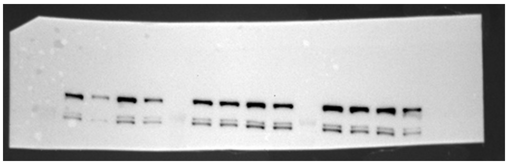
mTOR

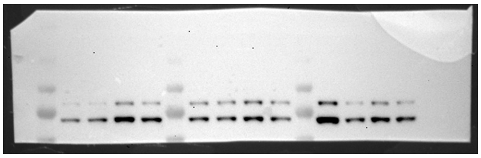
p70S6K (T389)

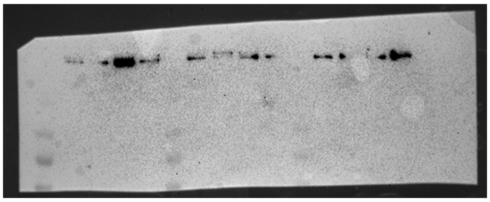
p70S6K (S371)

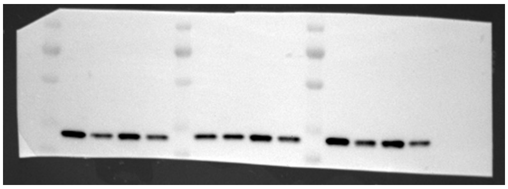
pS6R (S235/S236)

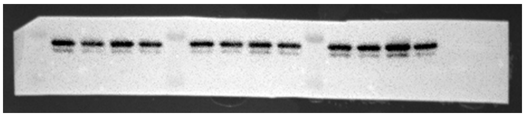
YB-1

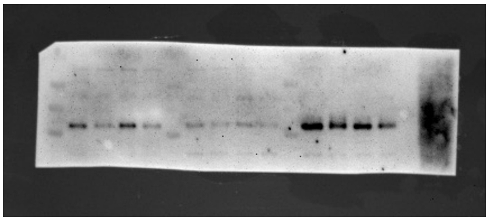
c-Myc

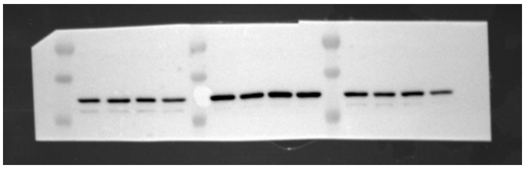
β actin

Figure 6B and Supplementary figure 9B

MDA-MB-231, MDA-MB-468 and SUM159

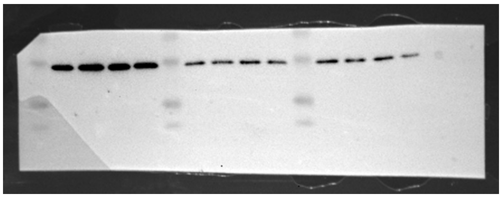

eIF6

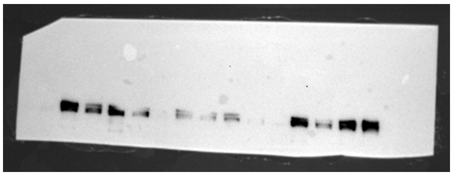

p-eIF4G

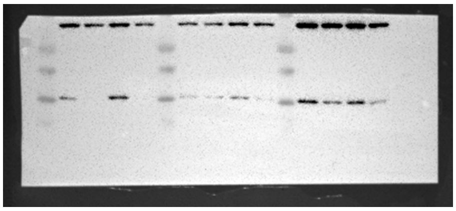

eIF4A

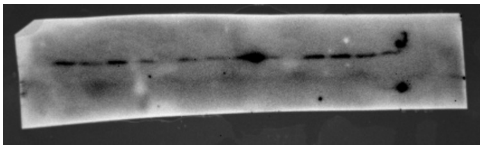

eIF1

MDA-MB-231, MDA-MB-468 and SUM159

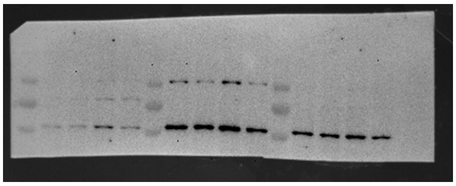

eIF5

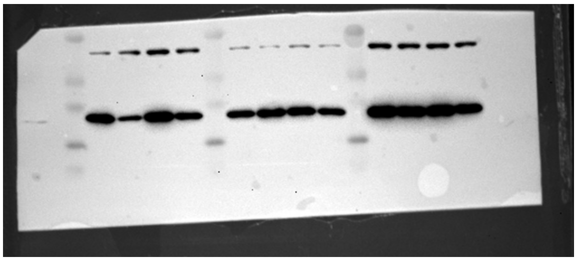

eIF4A1

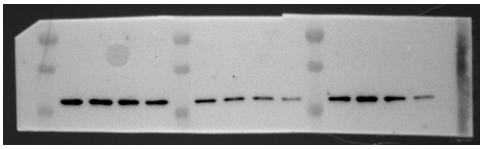

eIF3M

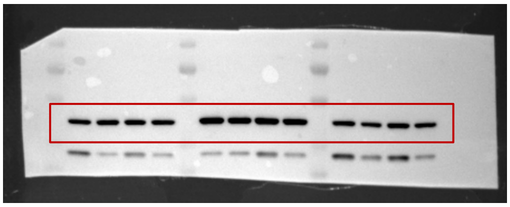

β actin

Figure 6C and Supplementary figure 9C

MDA-MB-231, MDA-MB-468 and SUM159

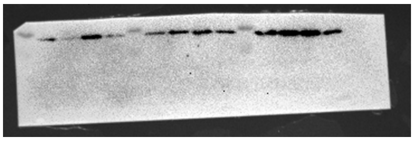

RPL36

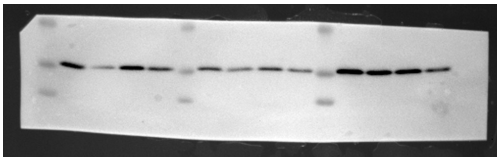

RPL32

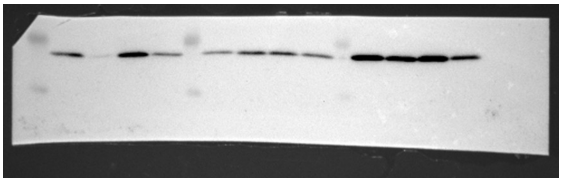

RPL15

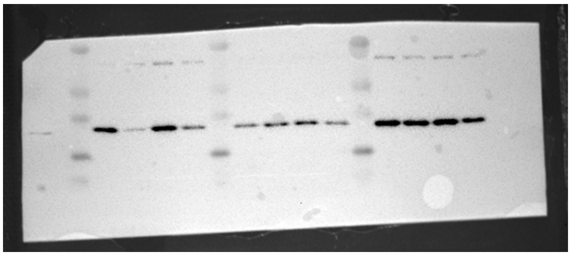

RPL9

MDA-MB-231, MDA-MB-468 and SUM159

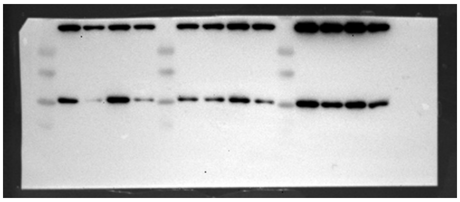

RPL35

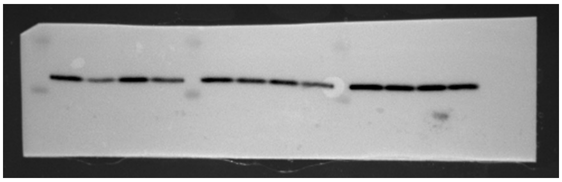

RPL23A

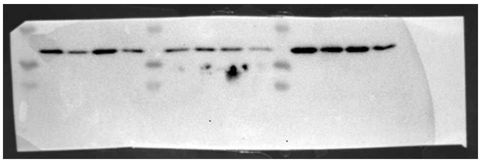

RPL11

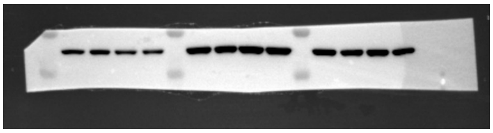

β actin

Figure 6D and Supplementary figure 9D

MDA-MB-231, MDA-MB-468 and SUM159

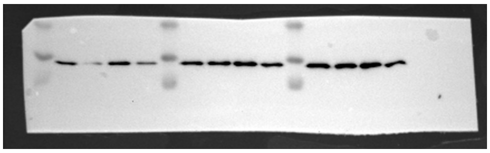
RPS20

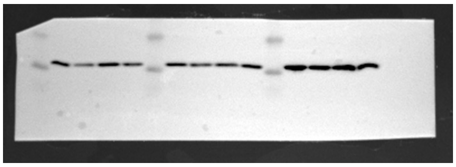
RPS15

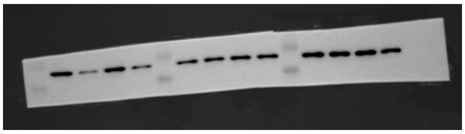
RPS3A

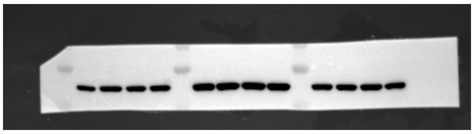
β actin

MDA-MB-231, MDA-MB-468 and SUM159

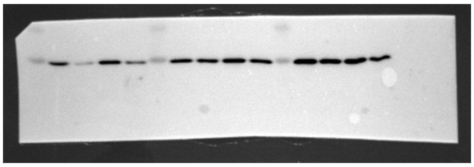
RPS16

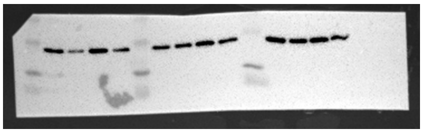
RPS9

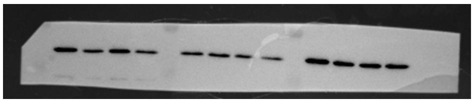
RPSA

Figure 7A (MDA-MB-231)

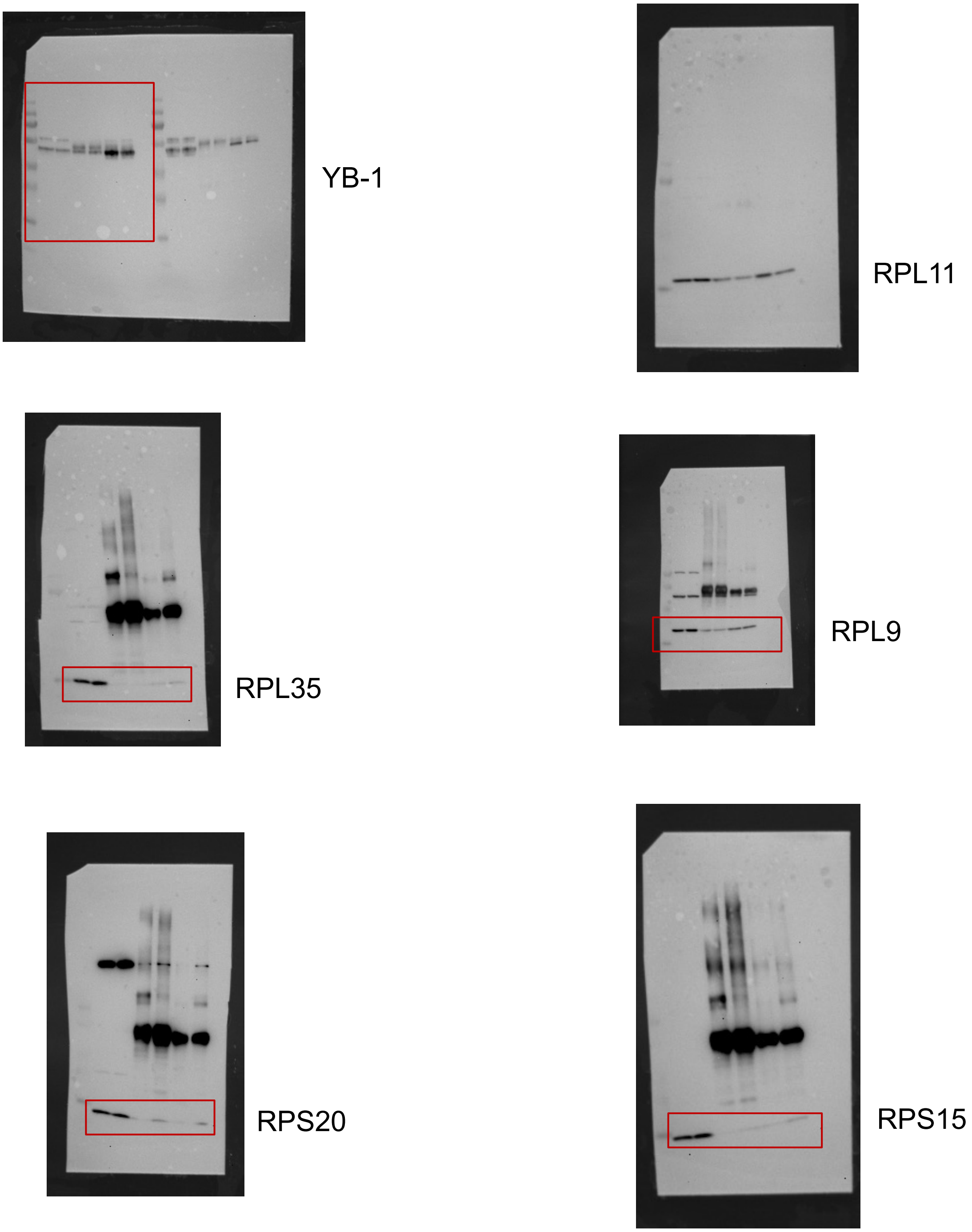

Figure 7A (MDA-MB-468)

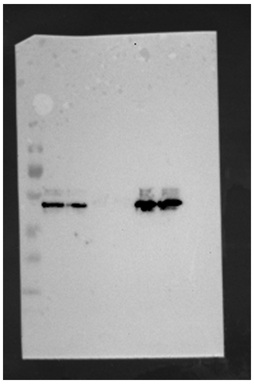

YB-1

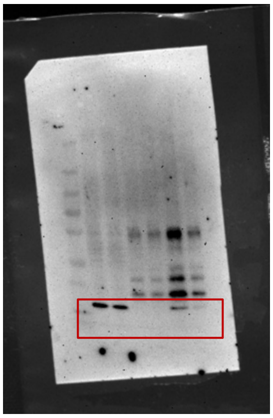

RPL11

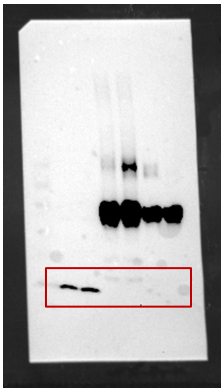

RPL35

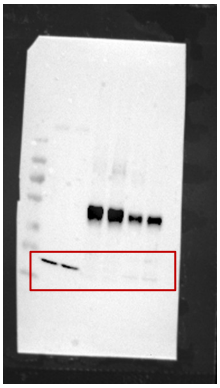

RPL9

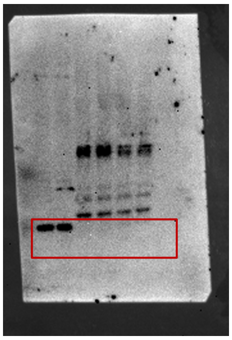

RPS20

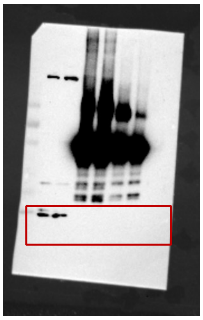

RPS15

Figure 7B (MDA-MB-231 and MDA-MB-468)

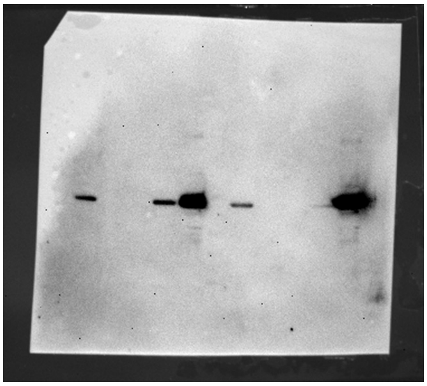

YB-1

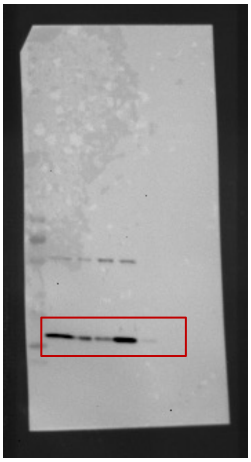

RPL11 (MDA-MB-231)

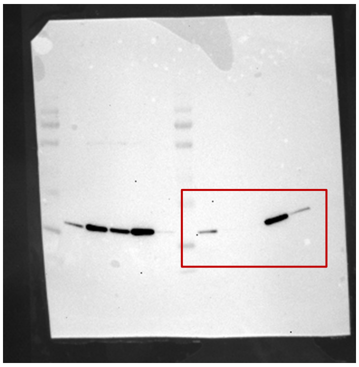

RPL11 (MDA-MB-468)

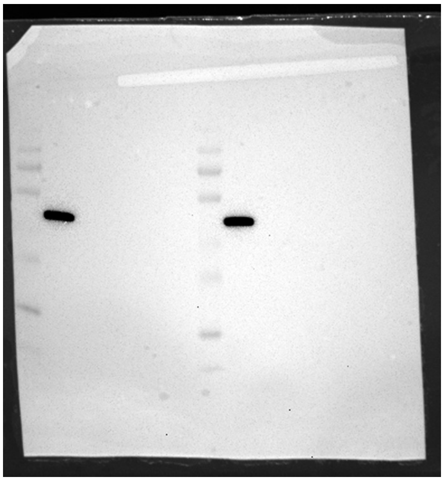

β actin

Figure 7C (MDA-MB-231)

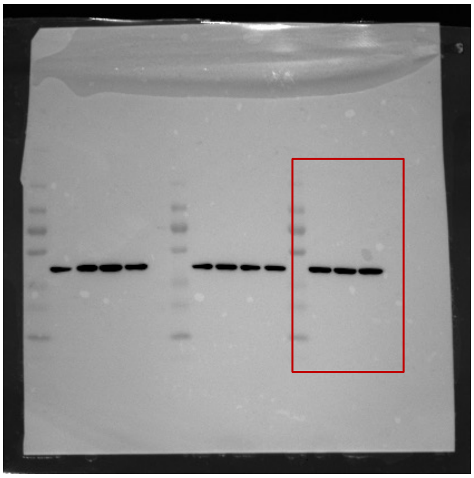

β actin

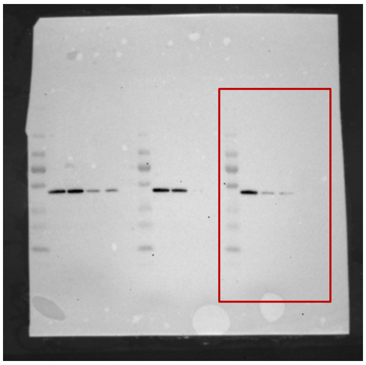

YB-1

Figure 7D (MDA-MB-231)

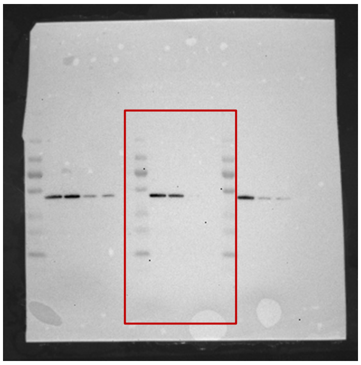

YB-1

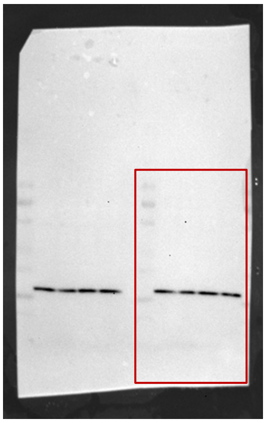

RPL11

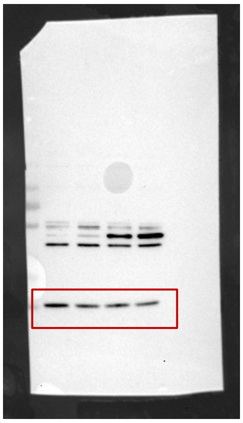

RPL32

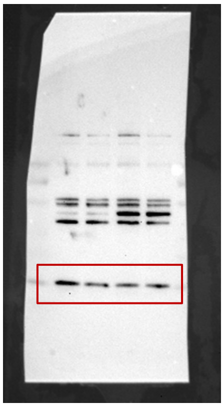

RPS20

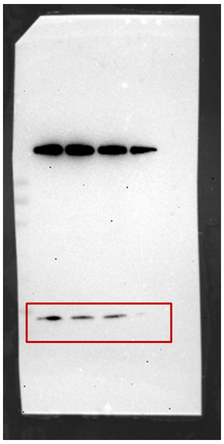

RPS15

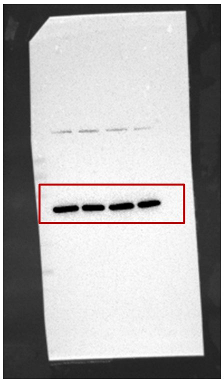

β actin

Figure 7D (MDA-MB-231 and MDA-MB-468)

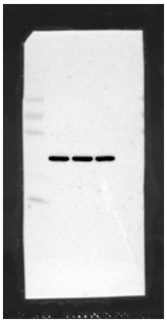

GAPDH (MDA-MB-231)

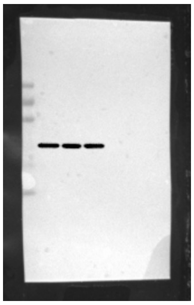

GAPDH (MDA-MB-468)

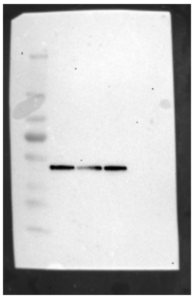

YB-1 (MDA-MB-231)

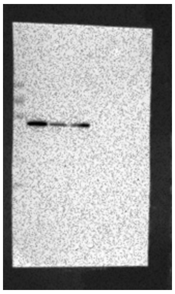

YB-1 (MDA-MB-468)

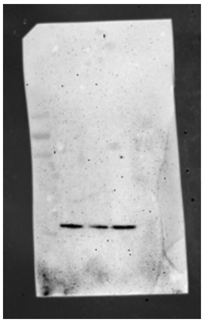

RPL11 (MDA-MB-231)

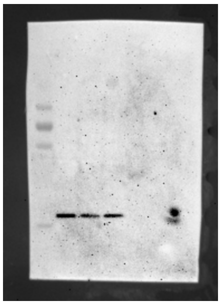

RPL11 (MDA-MB-468)

Figure 7H (CA1262-PDX-TNBC)

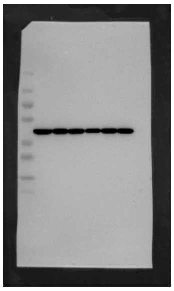

β actin (CA1262)

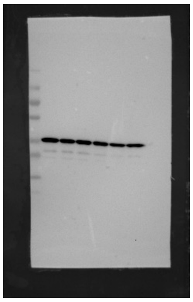

GAPDH (CA1262)

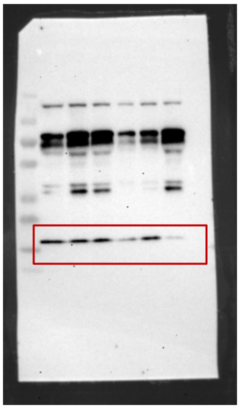

RPL11 (CA1262)

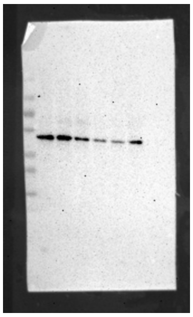

YB-1 (CA1262)

Figure 7I (MA2821-PDX-TNBC)

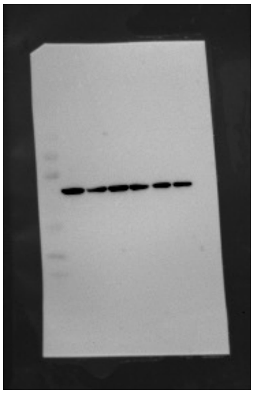

β actin (MA2821)

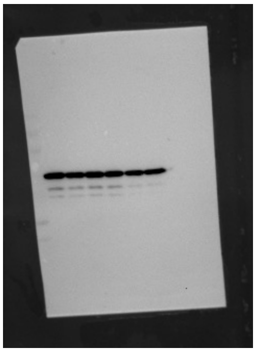

GAPDH (MA2821)

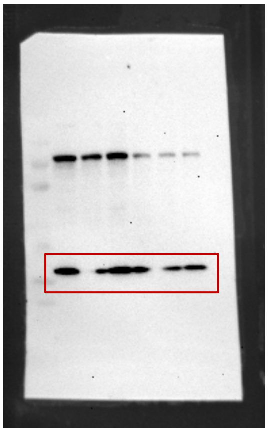

RPL11 (MA2821)

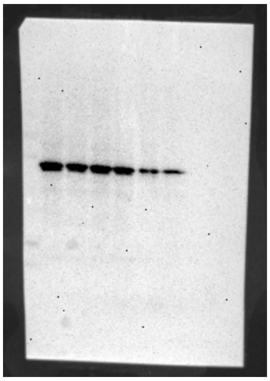

YB-1 (MA2821)

Supplementary figure 1B (MDA-MB-231 and MDA-MB-468)

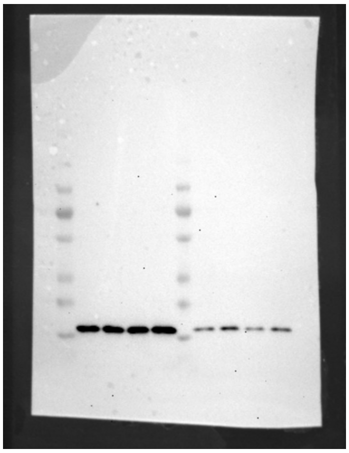

p21

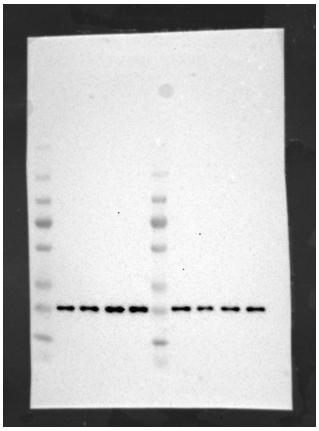

p27

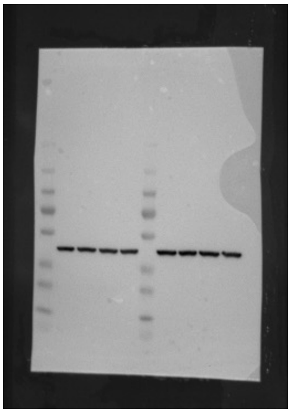

β actin

Supplementary figure 1E and 1F (MDA-MB-231 and MDA-MB-468)

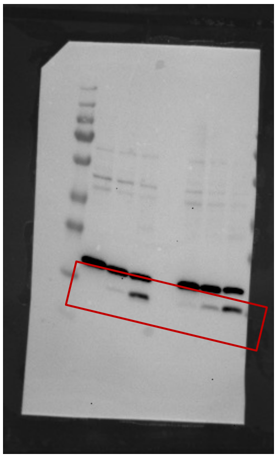

Bax

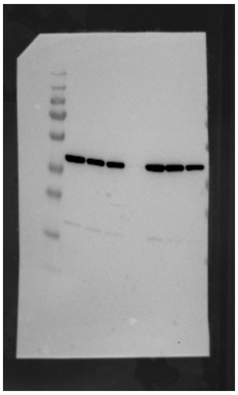

β actin

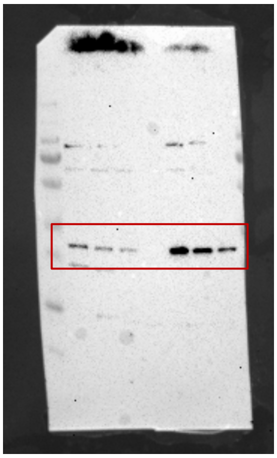

Bcl2

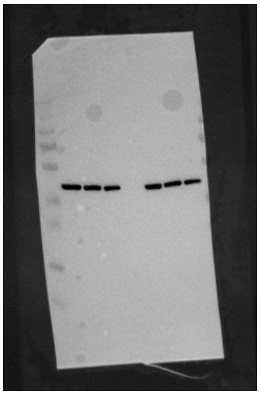

β actin

Supplementary figure 9E (MDA-MB-231 and MDA-MB-468)

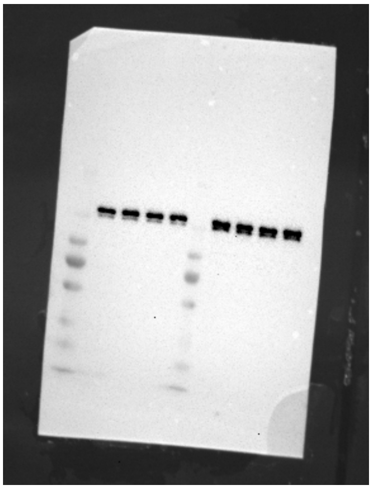
PERK

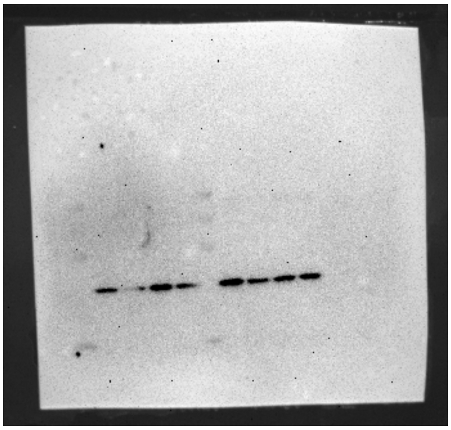
p-eIF2α

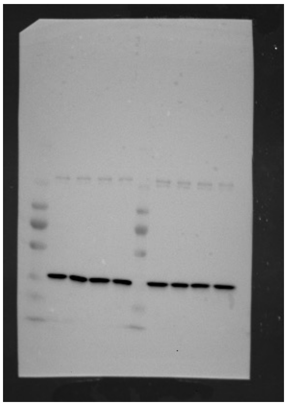
eIF2α

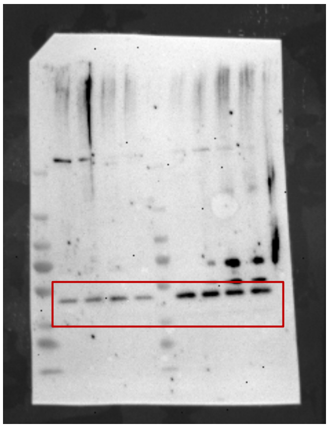
ATF4

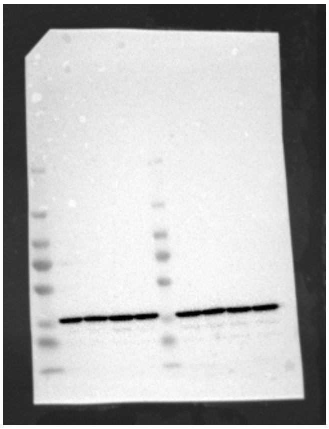
GAPDH
